# Latent Conditional Diffusion-Based Data Augmentation for Small-Sample Hyperspectral Prediction of Forest Soil Organic Carbon

**DOI:** 10.3390/s26175657

**Published:** 2026-09-05

**Authors:** Jian Tang, Weilin Li, Yuanyuan Shi, Yun Deng, Junyu Zhao

**Affiliations:** 1 Guangxi Forestry Laboratory, Guangxi Forestry Research Institute, Nanning 530002, China; 18378323910@163.com (J.T.); syyfly@163.com (Y.S.); 2 College of Computer Science and Engineering, Guilin University of Technology, Guilin 541004, China; 2120241286@glut.edu.cn (W.L.); 2002078@glut.edu.cn (Y.D.)

**Keywords:** hyperspectral sensor data, soil organic carbon, latent conditional diffusion, data augmentation, proximal sensing, forest soil, 1D-CNN, spectral regression

## Abstract

**Highlights:**

**What are the main findings?**

- A latent conditional diffusion framework was developed for hyperspectral sensor-based prediction of forest soil organic carbon under small-sample conditions.
- HsDDIM achieved the lowest MMD/FID values and improved the 1D-CNN validation performance to R^2^ = 0.91 and RMSE = 3.40 g kg^−1^.

**What are the implications of the main findings?**

- The proposed framework can improve AI-assisted hyperspectral sensing for forest soil carbon monitoring.
- SOC-conditioned latent diffusion provides a practical data augmentation strategy for limited labeled soil spectra.

**Abstract:**

Accurate forest soil organic carbon (SOC) monitoring is essential for forest soil quality assessment and carbon-sink evaluation. Visible-near-infrared (Vis–NIR) hyperspectral sensing provides rapid and information-rich measurements for SOC prediction, but obtaining sufficiently large labeled soil-spectral datasets remains difficult because field sampling, sample preparation, and reference SOC determination are labor and time intensive. This study developed a latent conditional diffusion-based data augmentation framework for SOC prediction from hyperspectral sensor data. A total of 248 forest red-soil samples from Guangxi, China, were measured using laboratory Vis–NIR reflectance spectroscopy over 350–2500 nm and divided by the Kennard-Stone algorithm into a 174-sample modeling set and a fixed 74-sample validation set. Four generative models, including VAE, GAN, WGAN-GP, and the proposed hyperspectral latent conditional denoising diffusion implicit model (HsDDIM), were evaluated using spectral visualization, t-SNE distributions, maximum mean discrepancy (MMD), Fréchet Inception Distance (FID), and downstream prediction performance. Unlike joint spectral-label generation, HsDDIM treats SOC as an external condition and generates spectra in the latent space under specified SOC conditions; the SOC condition itself is not generated by the diffusion process. Among the compared augmentation strategies, HsDDIM showed the closest distributional agreement with the real spectral samples according to MMD and FID, with values of 0.0806 and 0.5281, respectively. Without augmentation, FD1-SVR achieved the best validation result (R^2^ = 0.83, RMSE = 4.71 g kg^−1^). After 300% HsDDIM augmentation, 1D-CNN achieved R^2^ = 0.91, RPD = 3.39, and RMSE = 3.40 g kg^−1^. These results suggest that the SOC-conditioned latent DDIM framework can improve small-sample hyperspectral SOC prediction under the present fixed-validation protocol.

## 1. Introduction

Soil organic carbon (SOC) participates in nutrient cycling, soil aggregate formation, water retention, and terrestrial ecosystem carbon storage, and is therefore an important indicator for evaluating forest soil quality and ecological function [1,2]. Accurate and rapid acquisition of forest soil SOC content can support forest management, soil fertility assessment, and carbon-sink monitoring. Conventional SOC determination provides reliable reference measurements, but field sampling, sample preparation, and laboratory analysis are labor and time intensive, particularly when a large number of labeled samples is required for predictive modeling [3].

Visible-near-infrared (Vis–NIR) hyperspectral spectroscopy is attractive for soil-property assessment because it is rapid, non-destructive at the spectral measurement stage, and records continuous wavelength information that reflects multiple soil constituents [4,5]. SOC generally contributes to soil darkening and reduced overall reflectance, whereas iron oxides strongly affect the visible region and hydroxyl-bearing water and clay minerals contribute characteristic absorption features in the near- and shortwave-infrared regions. Thus, hyperspectral measurements provide rich information for SOC-related modeling even though individual spectral features are not uniquely controlled by SOC. In forest environments, complex terrain, dense vegetation, limited accessibility of sampling sites, and strong soil variability can restrict the number of spectra that are paired with reliable laboratory SOC reference values. The practical bottleneck is therefore not the size of the hyperspectral array itself, but the limited number of high-quality labeled soil spectra available for model development. Under such conditions, the combination of high-dimensional spectral inputs and limited labeled observations can make robust model calibration and generalization challenging [6,7].

Data augmentation is a practical strategy for alleviating limited-label problems. Conventional spectral augmentation methods, such as noise addition, shifting, and interpolation, are straightforward to implement and rely on manually specified perturbation rules [8]. Their suitability for preserving physically meaningful spectrum–SOC relationships therefore requires task-specific evaluation. GAN-based augmentation has been applied to soil spectral prediction problems [9,10], whereas adversarial training more generally can be sensitive to optimization settings and may suffer from mode collapse [11,12]. Diffusion models learn data distributions through progressive noise addition and reverse denoising using a stable noise-prediction objective [13,14]. However, diffusion directly in the original spectral space must simultaneously handle high dimensionality, spectral redundancy, and label matching. Compressing spectra into a low-dimensional representation while using SOC as an explicit conditioning signal therefore provides a potential route for reducing generative complexity while preserving the correspondence between generated spectra and SOC information [15,16].

Accordingly, this study used forest red soils in Guangxi as the research object and compared four learned data augmentation models, namely VAE, GAN, WGAN-GP, and HsDDIM, followed by SOC prediction using SVR, XGBoost, and 1D-CNN. HsDDIM is the methodological focus of this study. It integrates a spectral encoder, SOC label embedding, SOC-conditioned latent diffusion, DDIM-based reverse sampling, and conditional spectral reconstruction. Unlike a joint spectral-label diffusion process, the proposed implementation performs diffusion only in the 23-dimensional spectral latent space, while SOC is embedded as an external condition for denoising and decoding. The methodological contribution therefore lies in learning conditional spectral variability under specified SOC conditions rather than generating SOC as a stochastic diffusion variable. The objectives of this study are to (1) characterize the SOC content and hyperspectral reflectance patterns of forest red soils; (2) compare the spectral distributional agreement and downstream augmentation utility of pseudo-samples produced by the four learned augmentation strategies; (3) evaluate the effect of synthetic-sample proportion on SOC prediction under a common fixed-validation protocol; and (4) assess whether HsDDIM augmentation improves the performance of conventional machine-learning and 1D-CNN regression models.

## 2. Materials and Methods

### 2.1. Study Area and Sample Collection

The study area is located in a state-owned forest farm in Guangxi Zhuang Autonomous Region, China, within 22°49′–23°15′ N and 108°08′–108°53′ E. The region has a typical subtropical monsoon climate characterized by abundant precipitation, warm and humid conditions, and relatively stable vegetation cover. The forest farm covers approximately 890,000 mu (about 59,300 ha), with a forest coverage rate of 83.7% and a standing timber volume of approximately 7.0 million m^3^, and contains a mosaic of plantations and natural secondary forests.

**Figure 1:**
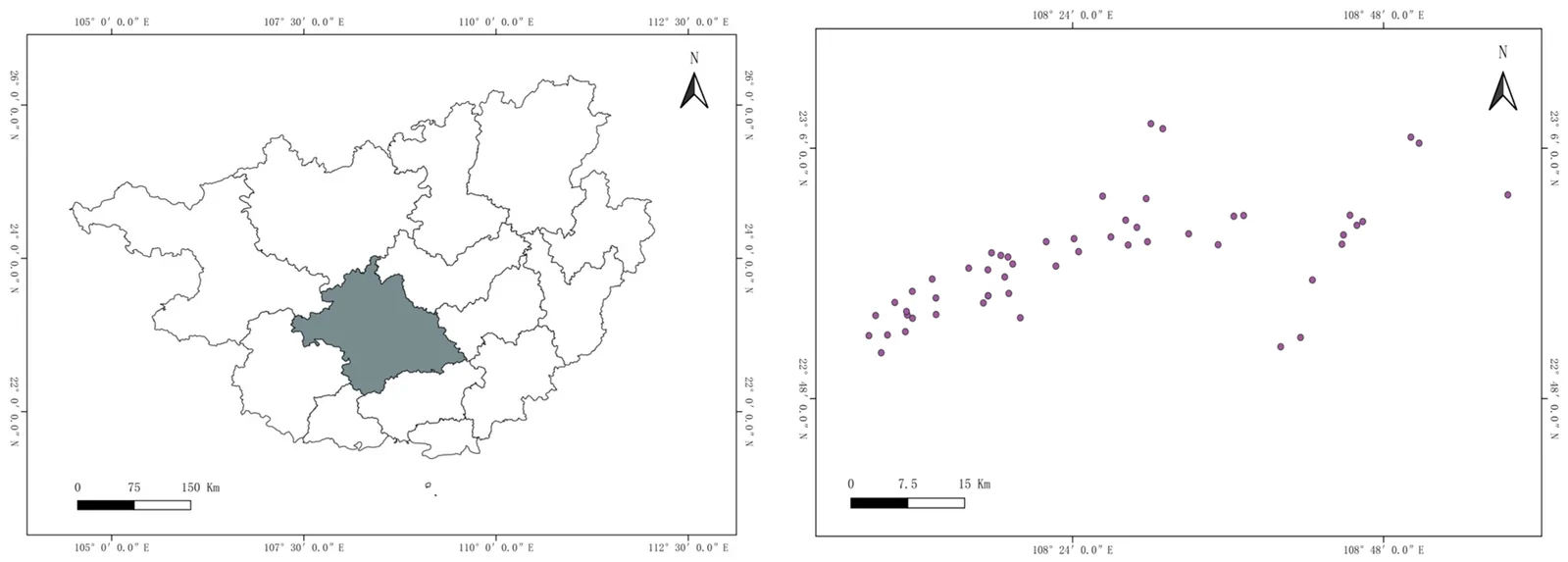
Study area and sampling distribution: (**Left**): location of the study area within Guangxi Province, China; (**right**): spatial distribution of soil sampling points within the forest farm.

The study area is dominated by subtropical red soil, with soil texture mainly ranging from loam to light clay and relatively strong water and nutrient retention capacity. The present study reports the pedological background at this regional soil-type level; a finer sample-level taxonomic classification was not used in the modeling analysis. The forest farm includes variation in topography, vegetation type, stand structure, and management practices. The SOC variability of the present Guangxi samples is assessed directly from the measured dataset in Section 3.1. Stable forest vegetation cover and high litter input provide a material basis for SOC accumulation, making this region a relevant setting for hyperspectral modeling of SOC in forest red soils [17]. The location of the study area and the spatial distribution of soil sampling points are shown in Figure 1.

### 2.2. Soil Sampling and Laboratory Measurement

To ensure sample representativeness, soil sampling was conducted before fertilization from March to April using a combination of grid layout and S-shaped sampling. Soil samples were collected from the 0–40 cm layer. This sampling depth was used to characterize integrated soil properties relevant to forest management and ecological assessment. A total of 248 soil samples were collected in this study. All samples were naturally air-dried indoors, cleaned of visible impurities, ground, and passed through a 2 mm sieve. All 248 samples had corresponding SOC reference measurements and hyperspectral reflectance records and were therefore included in the subsequent modeling analysis; no additional sample-level exclusion based on spectral outlier screening was performed.

Each sample was divided into two portions: one portion was used to determine SOC content by the potassium dichromate oxidation method [18], and the other was used for laboratory hyperspectral reflectance measurement. Spectral data were acquired using an ASD FieldSpec 4 Hi-Res spectroradiometer (ASD Inc., Boulder, CO, USA) over 350–2500 nm, with a spectral resolution of 1 nm. To reduce the effects of ambient light and instrumental drift, measurements were performed on a dark background and the instrument was periodically calibrated using a Spectralon^®^ diffuse reflectance panel (Labsphere, Inc., North Sutton, NH, USA) as the white reference. Each sample was scanned 10 times, and the mean reflectance was used as the final spectral curve for that sample.

### 2.3. Spectral Preprocessing and Sample Partitioning

To obtain representative modeling and validation subsets in the spectral feature space, the Kennard-Stone (KS) algorithm was used for sample partitioning. The KS algorithm selects representative samples based on inter-sample spectral distances and helps cover the major range of spectral variation in the dataset [19]. At an approximate ratio of 7:3, the 248 soil samples were divided into a 174-sample modeling set and a fixed 74-sample validation set. This 74-sample subset is hereafter referred to as the fixed validation set.

The 174-sample modeling set provided the real samples used for generative-model fitting, synthetic-sample construction, and predictive-model weight estimation. SVR and XGBoost hyperparameters were optimized by five-fold cross-validation within the modeling set. For 1D-CNN, early stopping was controlled by an internal validation subset drawn from the current modeling pool rather than by the fixed 74-sample set. The fixed 74-sample validation set was used for comparative evaluation of candidate preprocessing schemes, generative-model stages, augmentation ratios, and downstream prediction configurations. Model fitting, gradient-based optimization, and CNN early stopping remained restricted to the modeling data. Therefore, the reported metrics represent fixed-validation performance rather than performance on a strictly untouched external test set, and some selection bias associated with repeated validation should be acknowledged [20].

After dataset partitioning, the raw hyperspectral data were preprocessed following a commonly used spectral preprocessing procedure. Edge bands with low signal-to-noise ratios, including 350–399 nm and 2401–2500 nm, were first removed. Savitzky–Golay (SG) smoothing was then applied to reduce random spectral noise, with a window length of 23 and a polynomial order of 3 [21]. To reduce spectral baseline drift caused by differences in soil particle size, surface roughness, and scattering effects, multiplicative scatter correction (MSC) was further applied to the SG-smoothed spectra [22]. Following the commonly used MSC procedure, the mean spectrum of the modeling samples was used as the fixed reference spectrum. For the i-th sample spectrum $x_{i}(\lambda )$, a linear regression relationship was established between $x_{i}(\lambda )$ and the reference mean spectrum $\bar{x}(\lambda )$:

(1) $$x_{i}(\lambda )=a_{i}+b_{i}\bar{x}(\lambda )+e_{i}(\lambda )$$

Based on the estimated additive offset and multiplicative scattering coefficients, the sample spectrum was corrected as follows:

(2) $$x_{i}^{MSC}(\lambda )=\frac{x_{i}(\lambda )-a_{i}}{b_{i}}$$

where λ denotes wavelength; $a_{i}$ and $b_{i}$ are the additive offset coefficient and multiplicative scattering coefficient of the i-th sample spectrum relative to the reference spectrum, respectively; $e_{i}(\lambda )$ is the residual term; and $x_{i}^{MSC}(\lambda )$ denotes the reflectance spectrum after MSC correction.

After basic preprocessing, multiple spectral transformation features were further constructed to enhance local absorption information and spectral variation features related to SOC content [23]. These included the non-derivative baseline spectrum (denoted Raw), first-order derivative spectrum (FD1), second-order derivative spectrum (FD2), and 1.2-, 1.4-, 1.6-, and 1.8-order fractional-order derivative spectra (FOD). FD1 and FD2 were calculated using Savitzky–Golay differentiation with the same window length (23) and polynomial order (3) used for smoothing. Common definitions of fractional-order derivatives include the Grünwald–Letnikov (GL), Riemann–Liouville, and Caputo formulations [24]. In this study, the GL definition was used to perform fractional-order derivative transformation of spectral reflectance, and its expression is as follows:

(3) $$\frac{d^{a}R(\lambda )}{d\lambda^{a}}=\underset{h\to 0}{\lim}\frac{1}{h^{a}}\sum_{m=0}^{\frac{\lambda -\lambda_{0}}{h}}(-1{)}^{m}\frac{\Gamma (a+1)}{m!\Gamma (a-m+1)}R(\lambda -mh)$$

where a denotes the fractional derivative order; λ denotes the current wavelength; $\lambda_{0}$ denotes the initial wavelength of the spectrum; R(λ) denotes the spectral reflectance at wavelength λ; h is the spectral sampling interval; m is the discrete accumulation index; and Γ(⋅) is the Gamma function. In this study, 1.2-, 1.4-, 1.6-, and 1.8-order FOD spectra were constructed and used together with Raw, FD1, and FD2 spectra for subsequent modeling analyses. The GL step size h was set to 1 nm, corresponding to the original spectral sampling interval used for the derivative calculation.

**Figure 2:**
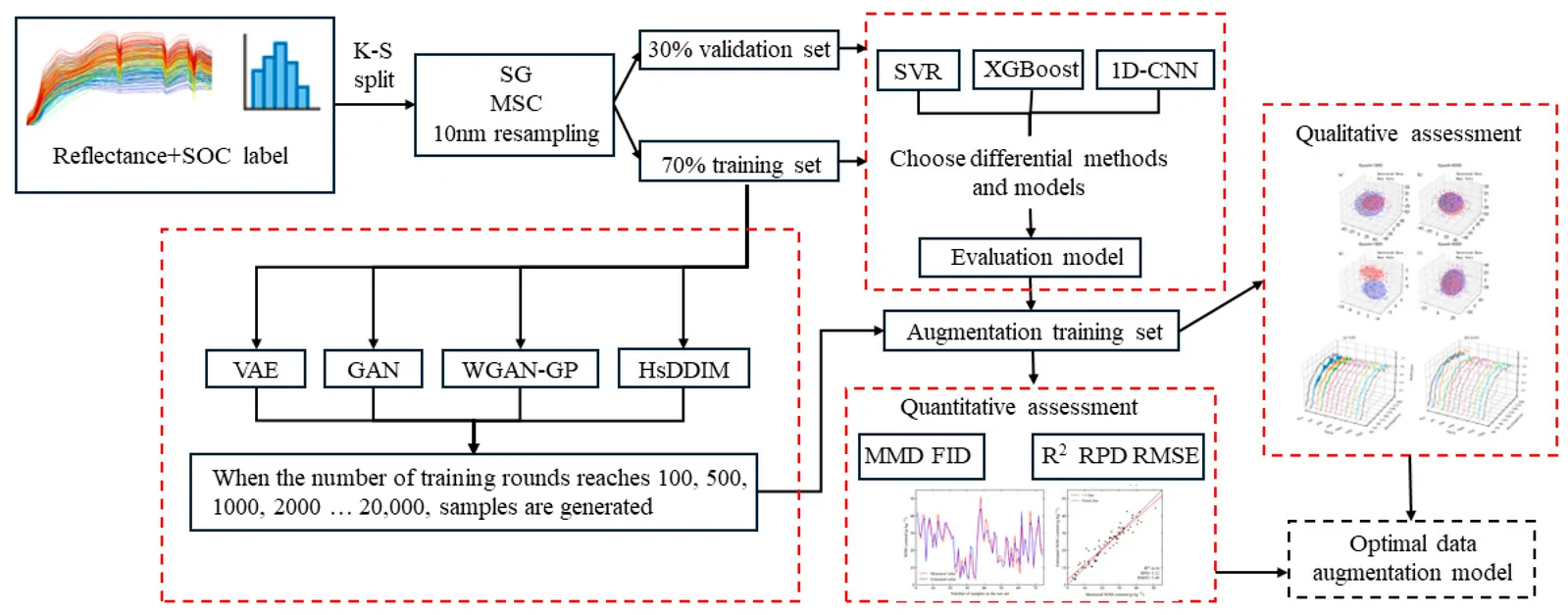
Overall experimental workflow, including spectral preprocessing, data augmentation, pseudo-sample quality evaluation, and SOC prediction modeling. The fixed validation set was used for comparative evaluation of candidate configurations, whereas model fitting was performed using the modeling data. Arrows indicate the direction of data and model flow, while the red dashed boxes group the main functional modules for data augmentation, prediction-model evaluation, and qualitative/quantitative assessment. Colors are used only for visual distinction and do not encode quantitative information.

Finally, to reduce band redundancy and standardize the input dimensionality for subsequent prediction and generative models, the preprocessed spectra were resampled at 10 nm intervals. Each sample ultimately retained 201 spectral bands as model inputs. This processing workflow preserved the main spectral morphology while reducing random noise and redundant bands, thereby providing a unified spectral feature space for subsequent SOC prediction modeling and generative data augmentation. For subsequent latent-space and distance-based analyses, the 201-dimensional spectral variables were standardized bandwise using z-score normalization. The joint spectral–SOC normalization used by VAE, GAN, and WGAN-GP and the model-specific standardization used by the prediction models are described in Section 2.4 and Section 2.5, respectively. The overall experimental workflow, including spectral preprocessing, data augmentation, pseudo-sample quality evaluation, and SOC prediction modeling, is summarized in Figure 2.

### 2.4. Data Augmentation Models and HsDDIM Architecture

To compare learned generative strategies for hyperspectral data augmentation in forest red soils, this study constructed four models: VAE, GAN, WGAN-GP, and HsDDIM [11,12,13,14,15,16]. Generated spectral quality, statistical distributional agreement, and downstream predictive utility were used as common evaluation criteria without assuming that the four models implemented identical probabilistic generation tasks. For VAE, GAN, and WGAN-GP, each training sample was represented as a 202-dimensional joint spectral–SOC vector consisting of the 201 preprocessed spectral bands and one SOC value. The spectral variables and SOC were normalized jointly before generative-model training. These three baseline models therefore learned and generated the joint spectral–SOC representation; after inverse transformation, the first 201 variables were used as the synthetic spectrum and the final variable as its generated SOC label. In contrast, HsDDIM does not generate SOC. It treats SOC as an external conditioning signal and generates only the spectrum under the specified SOC condition.

To make the baseline implementation reproducible, the VAE, GAN, and WGAN-GP architectures followed Xia et al. [9], with only the joint input/output dimensionality adapted to 202 variables for the present 201-band spectrum-plus-SOC representation. The generator/decoder used a 100-dimensional latent/noise input followed by dense layers with 32, 64, and 128 units, ReLU activations, batch normalization, and a final 202-unit Tanh output layer. The encoder/discriminator used two Conv1D blocks with 32 and 64 channels, respectively, each followed by LeakyReLU (0.2) and dropout (0.3), then a flattening layer and a 128-unit dense layer. GAN used a Sigmoid scalar output, whereas the WGAN-GP critic used a linear scalar output without Sigmoid; the VAE encoder additionally used mean and variance heads to parameterize the latent distribution. The three baselines were optimized with Adam [25] using an initial learning rate of 0.0002 and moment coefficients of 0.5 and 0.9 and were trained for up to 20,000 iterations. For WGAN-GP, the critic was updated five times before each generator update. These reference-based baseline settings were kept fixed rather than re-optimized specifically for the present dataset. HsDDIM used the separate AdamW-based latent-diffusion configuration described in Section 2.4.4.

#### 2.4.1. Variational Autoencoder (VAE)

**Figure 3:**
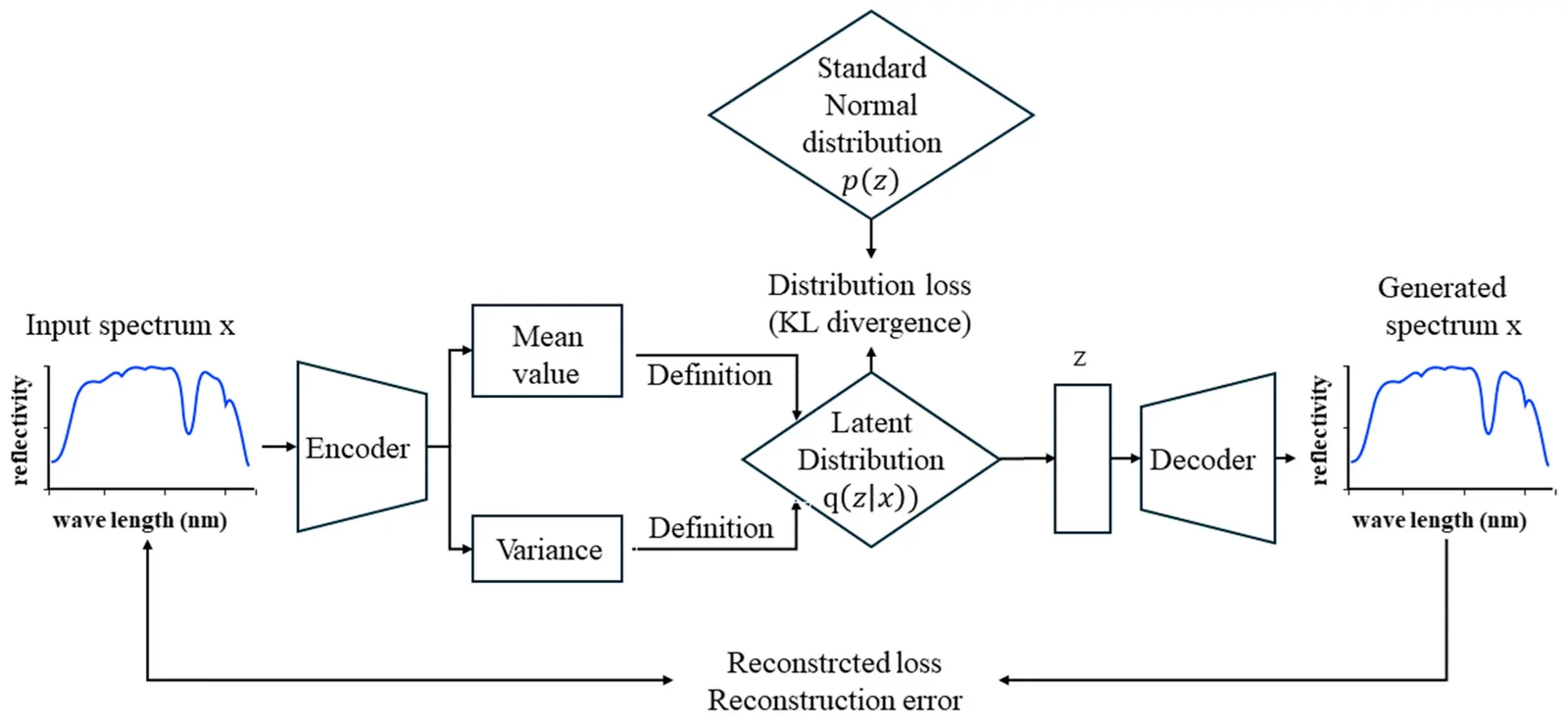
Encoding, latent-variable sampling, and decoding reconstruction process of the VAE baseline. The schematic shows the spectral component for visualization; in the implemented baseline, the corresponding SOC variable was included in the jointly normalized 202-dimensional input and reconstructed output. Arrows indicate the encoding and decoding pathways, the construction and sampling of the latent distribution, and the connections used to compute the KL-divergence and reconstruction losses.

The variational autoencoder (VAE) consists of an encoder and a decoder (Figure 3) [15]. In the baseline implementation used here, the encoder maps the jointly normalized 202-dimensional spectral–SOC vector into a latent distribution and outputs its mean and variance parameters. The decoder reconstructs the same joint representation from a sampled latent variable. Thus, the VAE baseline generates spectrum and SOC together rather than assigning an SOC value to a generated spectrum afterward.

The training objective of VAE consists of a reconstruction term and a KL divergence term. The reconstruction term constrains the difference between reconstructed and real joint spectral–SOC samples, while the KL divergence constrains the posterior distribution of the latent variables toward the predefined prior distribution. The VAE loss function is expressed as

(4) $$L_{VAE}=\frac{1}{n}\sum_{i}∥x_{i}-{\hat{x}}_{i}{∥}_{2}^{2}+D_{KL}\left(q_{\phi }\left(z|x\right)\;∥\;p\left(z\right)\right)$$

Given an input joint sample x, the encoder estimates the mean μ and standard deviation σ of the latent distribution and uses the reparameterization trick to sample z, with random noise ε drawn from a standard normal distribution. The decoder maps z back to the joint data space to obtain the reconstruction $\hat{x}$. Here, $q_{\phi }\left(z|x\right)$ denotes the learned latent posterior and $p\left(z\right)$ denotes the standard normal prior. The reconstruction term uses mean squared error (MSE). After inverse transformation of a generated joint vector, its first 201 variables are interpreted as the synthetic spectrum and its final variable as the generated SOC label.

#### 2.4.2. Generative Adversarial Network (GAN)

**Figure 4:**
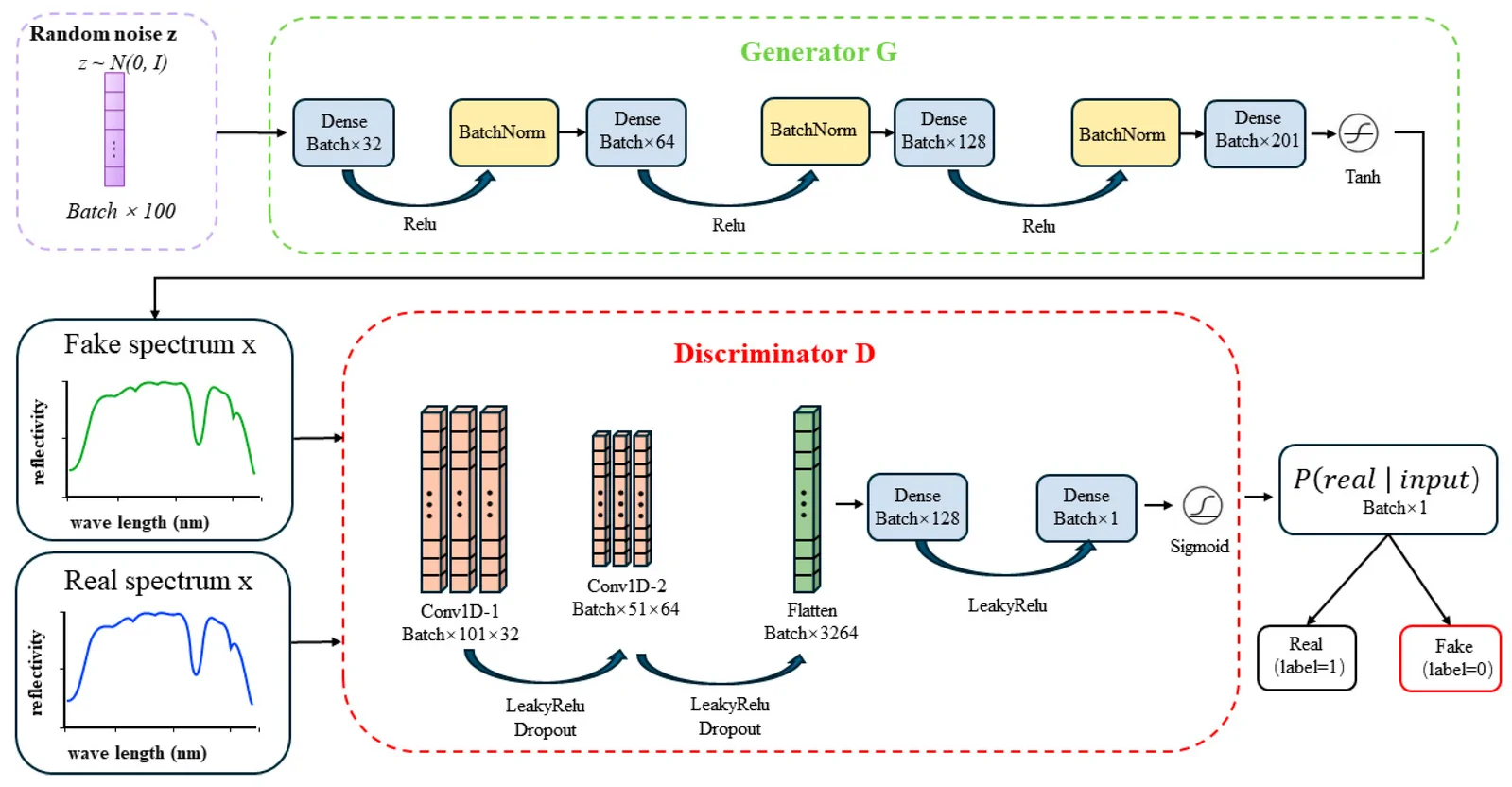
Basic GAN framework. The schematic displays the 201-band spectral component; in the implemented joint spectral–SOC baseline, one SOC variable was appended, giving a 202-dimensional generated vector. Arrows indicate the data flow from random noise through the generator to the generated spectrum and from the real/generated spectra through the discriminator to the real/fake classification output. Colors and dashed boxes are used to visually distinguish the generator, discriminator, input, and output components and do not encode quantitative information. Ellipses within the vector representations indicate omitted intermediate elements for compact visualization and do not represent omitted network components.

The generative adversarial network (GAN) consists of two subnetworks: a generator G and a discriminator D (Figure 4) [11]. In this study, the generator takes random noise z drawn from a prior distribution as input and generates a jointly normalized spectral–SOC pseudo-sample. The discriminator receives both real and generated joint spectral–SOC vectors and outputs the probability that the input originates from the real distribution. The generator and discriminator are optimized adversarially so that the generated joint distribution progressively approaches the real joint distribution.

The training process of GAN can be formulated as a minimax optimization problem, with the objective function expressed as

(5) $$\underset{G}{min}\underset{D}{max}V\left(D,G\right)=E_{x∼p_{r}}\left[logD\left(x\right)\right]+E_{z∼p_{z}}\left[log\left(1-D\left(G\left(z\right)\right)\right)\right]$$

where x denotes a real joint spectral–SOC sample, z denotes a random variable sampled from the prior noise distribution $p_{z}$, $G\left(z\right)$ denotes the generated joint pseudo-sample, $D\left(\cdot \right)$ denotes the discriminator output, and $p_{r}$ denotes the real joint-data distribution. After inverse transformation, each generated joint vector is separated into its 201-band synthetic spectrum and generated SOC value for downstream augmentation.

#### 2.4.3. Wasserstein GAN with Gradient Penalty (WGAN-GP)

Wasserstein GAN with gradient penalty (WGAN-GP) is an improved generative adversarial model developed on the basis of traditional GAN [12,26]. Traditional GANs usually construct adversarial losses using the probability values output by the discriminator. However, when the real and generated sample distributions have limited overlap, the discriminator can quickly distinguish the two sample types, resulting in insufficient gradient information for the generator and thus affecting training stability [27]. WGAN-GP uses the Wasserstein distance to measure the discrepancy between the real and generated distributions, providing the generator with a more continuous and stable optimization signal. In the present baseline implementation, both real and generated samples were the jointly normalized 202-dimensional spectral–SOC vectors described above.

In WGAN-GP, the discriminator is usually referred to as the critic. Unlike the discriminator in a traditional GAN, which outputs the probability that a sample belongs to the real distribution, the critic outputs a continuous real-valued score to evaluate the relative difference between the input sample and the real/generated distributions. Therefore, the final layer of the critic in WGAN-GP does not use a Sigmoid activation function. To ensure effective estimation of the Wasserstein distance, the critic must satisfy the K-Lipschitz continuity constraint, which is expressed as

(6) $$K-Lipschitz=\left|f(x_{1})-f(x_{2})\right|\leq K\left|x_{1}-x_{2}\right|$$

where $x_{1}$ and $x_{2}$ denote any two input samples, $f(x_{1})$ and $f(x_{2})$ denote the corresponding outputs of the critic, and K is the Lipschitz constant. When K=1, the critic satisfies the 1-Lipschitz continuity constraint, meaning that the magnitude of its output variation is bounded by the magnitude of the input variation, thereby avoiding excessive local gradient fluctuations in the critic.

Under the Lipschitz continuity constraint, the Wasserstein distance between the real sample distribution and the generated sample distribution can be expressed as

(7) $$Wasserstein\;Loss=\frac{1}{K}\underset{{\left\|D\right\|}_{L}\leq K}{\sup}E_{x∼p_{r}}\left[D(x)\right]-E_{x∼p_{g}}\left[D(G(x))\right]$$

where D(⋅) denotes the output function of the critic, $p_{r}$ denotes the real sample distribution, $p_{g}$ denotes the generated sample distribution, and ${\left|D\right|}_{L}\leq K$ indicates that the critic satisfies the K-Lipschitz continuity constraint. This objective estimates the distance between the two distributions by maximizing the difference between the scores of real and generated samples, thereby providing a more stable training direction for the generator.

Early WGANs usually used weight clipping to approximately satisfy the Lipschitz constraint, but weight clipping may limit the representational capacity of the critic and affect model convergence. WGAN-GP introduces a gradient penalty term to constrain the gradient norm of the critic, maintaining smooth variation in the transition region between real and generated samples. Its loss function can be expressed as

(8) $$L_{D}=E\left[D\left(\tilde{x}\right)\right]-E\left[D\left(x\right)\right]+\lambda E\left[{\left(∥\nabla_{\hat{x}}D\left(\hat{x}\right){∥}_{2}-1\right)}^{2}\right],\;\;L_{G}=-E\left[D\left(\tilde{x}\right)\right]$$

where $\tilde{x}$ denotes a generated sample, $\hat{x}$ denotes a sample obtained by linear interpolation between a real sample and a generated sample, $p_{\hat{x}}$ is the interpolated sample distribution, and λ is the weight coefficient of the gradient penalty term. The first two terms in the formula measure the Wasserstein distance between the real and generated distributions, whereas the last term is the gradient penalty term, which constrains the gradient norm of the critic at the interpolated samples to be close to 1. Through the joint constraint of the Wasserstein distance and gradient penalty, WGAN-GP can alleviate gradient instability and mode collapse that may occur during traditional GAN training, thereby improving the stability and distributional consistency of generated samples.

#### 2.4.4. HsDDIM Latent Conditional Diffusion Implicit Model

**Figure 5:**
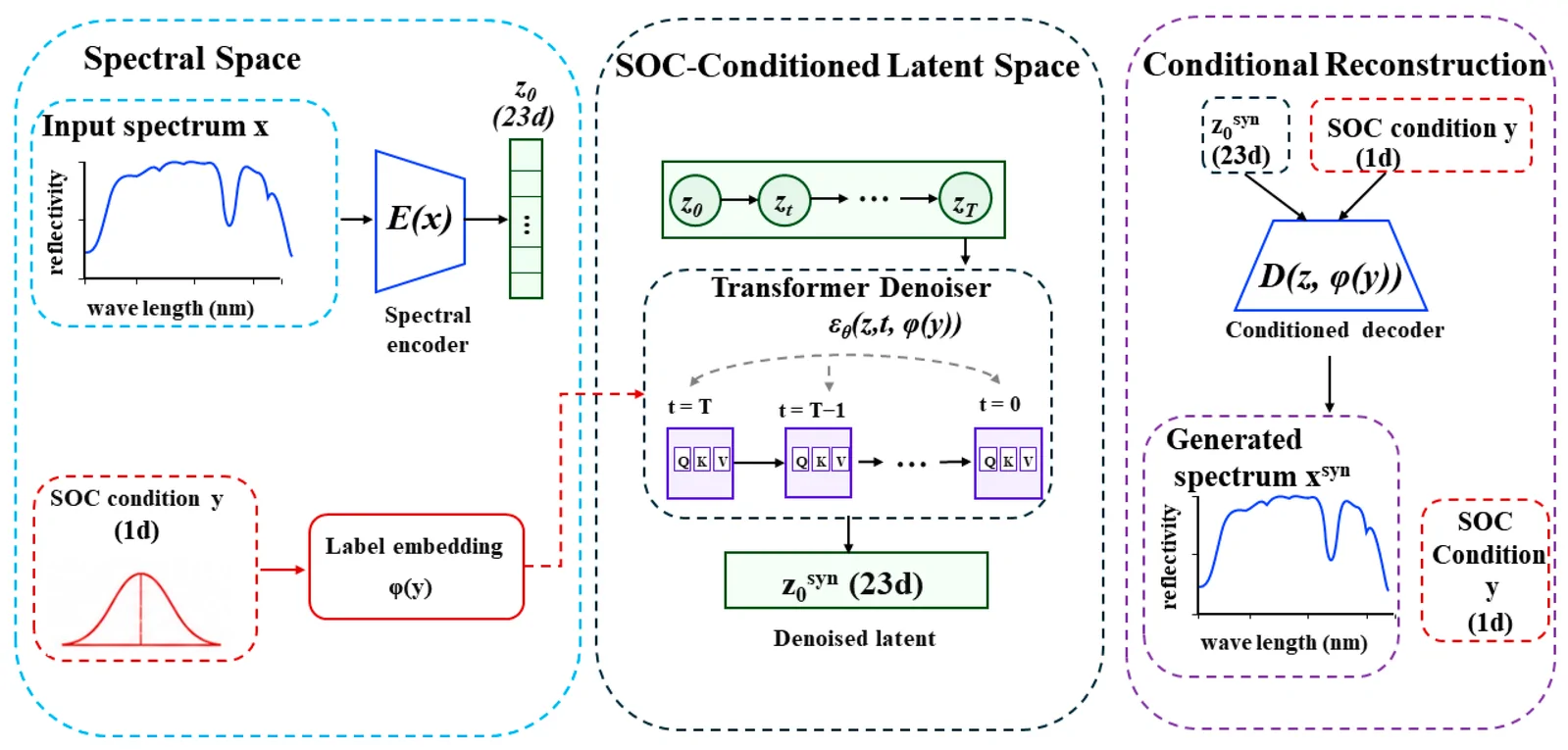
Architecture of the HsDDIM SOC-conditioned latent denoising diffusion implicit model. Spectrum x is encoded into a 23-dimensional spectral latent vector z_0_. SOC y is embedded as an external condition and combined with the diffusion-time embedding to guide the Transformer denoiser. DDPM-style noise prediction and DDIM sampling operate only in the spectral latent space; the decoder reconstructs x^syn^ from the sampled latent vector under the specified SOC condition. The SOC value is not diffused or generated. Arrows indicate information flow, colored dashed outlines distinguish the three functional modules, and ellipses denote intermediate diffusion timesteps and latent states omitted for compact visualization; no model components are omitted, and colors do not encode quantitative information.

HsDDIM is an SOC-conditioned latent denoising diffusion implicit model for hyperspectral data augmentation, and its implementation architecture is shown in Figure 5. Diffusion models have also been explored for hyperspectral data modeling, where iterative denoising is used to characterize distributions in high-dimensional and redundant spectral data [28]. The spectral encoder–decoder used to establish the latent space was implemented as a conditional variational autoencoder (CVAE): the encoder compresses each preprocessed spectrum into a spectral latent representation, while SOC is supplied to the decoder through a separate conditioning embedding. SOC is not concatenated with the spectral latent vector as an additional diffusion dimension; instead, it guides both the Transformer denoiser and the decoder. Thus, the diffusion state contains spectral latent variables only, while SOC remains an external conditioning variable. Operationally, HsDDIM models a conditional spectral distribution given SOC rather than a joint diffusion over spectra and SOC labels [13,14,16].

The CVAE used a 1024-unit hidden representation, a 23-dimensional spectral latent vector, and a 32-dimensional SOC embedding in the conditional decoder. It was trained for 5000 epochs using AdamW with a learning rate of 1 × 10^−3^ and weight decay of 1 × 10^−5^. The configured batch size was 512; because the modeling set contained 174 samples, CVAE updates were effectively full-batch. The reconstruction term used MSE and the KL-divergence term was weighted by 1 × 10^−3^. Two weak auxiliary spectral constraints were retained in the physical-CVAE pipeline: an output-range penalty with weight 0.05 and a local absorption-region penalty with weight 0.02 around the 1400, 1900, and 2200 nm regions (±20 nm). These settings were kept unchanged in the latent-dimensionality comparison described below.

The spectral latent dimension was set to 23 based on preliminary model-development experiments. To quantitatively document this choice, the latent-dimensionality comparison was repeated using candidate dimensions of 8, 16, 23, and 32 while all other preprocessing and CVAE training settings were held constant. The fixed-validation reconstruction RMSE values were 0.14094, 0.11730, 0.09821, and 0.10067, respectively, with the 23-dimensional setting yielding the lowest reconstruction error. PCA of the standardized 201-dimensional SG first-derivative spectra in the 174-sample modeling set further showed that 22 principal components were sufficient to exceed 99.9% cumulative explained variance, while 23 components retained 99.9235%. Therefore, 23 dimensions were retained as a compact spectral bottleneck that provided the best observed validation reconstruction performance among the evaluated settings while remaining close to the intrinsic dimensionality indicated by PCA. Full results are provided in Supplementary Table S2.

Given a hyperspectral sample x and its measured SOC value y, the spectral encoder $E\left(\cdot \right)$ produces $z_{0}=E\left(x\right)$, where $z_{0}\in R^{23}$. A separate label network maps y to a conditioning representation $\phi \left(y\right)$. In the decoder, the 23-dimensional spectral latent vector is combined with a label embedding to reconstruct a spectrum under the specified SOC condition. The SOC label itself is neither noised nor generated by the diffusion process; it serves only as the condition that anchors spectral generation.

(9) $$z_{0}=E\left(x\right),\;\;z_{0}\in R^{23}$$

The conditional reconstruction can be summarized as $x^{syn}=D\left(z_{0}^{syn},\phi \left(y\right)\right)$, where $z_{0}^{syn}$ is a synthetic spectral latent vector produced by reverse diffusion and $D\left(\cdot \right)$ is the spectral decoder. This design makes the generated spectrum explicitly dependent on the specified SOC condition without treating SOC as part of the stochastic diffusion state.

Forward diffusion is applied only to the spectral latent vector. For diffusion step t, the latent distribution follows the standard DDPM forward process. The implementation used T=50 diffusion steps and a linear variance schedule with $\beta_{1}=1\times 10^{-4}$ and $\beta_{T}=2\times 10^{-2}$.

(10) $$q\left(z_{t}|z_{0}\right)=N\left(\sqrt{{\bar{\alpha }}_{t}}z_{0},\left(1-{\bar{\alpha }}_{t}\right)I\right)$$

As t increases, Gaussian noise progressively perturbs the spectral latent representation while the SOC condition remains unchanged. Consequently, the stochastic diffusion process models variation in the spectral latent distribution, whereas SOC provides a fixed conditioning signal that guides the reverse denoising trajectory.

The reverse process uses a ConditionalTransformerDenoiser, drawing on self-attention-based Transformer modeling and diffusion-Transformer designs [29,30]. The 23 dimensions of $z_{t}$ are treated as a sequence of 23 latent tokens and projected to $d_{model}=128$. Diffusion time is represented by a 128-dimensional sinusoidal embedding followed by a two-layer SiLU MLP. The scalar SOC value is mapped through a label embedding network, and the time and label embeddings are added to form the conditioning vector that is broadcast to all latent-token positions. The Transformer contains three encoder layers, eight attention heads, a 512-dimensional feed-forward subnetwork, dropout of 0.1, and GELU activation.

(11) $$L_{diff}=E\left[∥\epsilon -\epsilon_{\theta }\left(z_{t},t,y\right){∥}_{2}^{2}\right]$$

The denoiser is optimized using a DDPM-style noise-prediction objective, where ε is the Gaussian noise added to $z_{0}$ and $\varepsilon_{\theta }\left(\cdot \right)$ is the conditional Transformer prediction. For the latent denoiser, AdamW optimization was used with a learning rate of 1 × 10^−4^, weight decay of 1 × 10^−5^, and the default AdamW moment coefficients (0.9, 0.999). The main training routine used a fixed learning rate and a maximum of 20,000 iterations with random seed 42. With 174 real modeling samples, the configured batch limit resulted in full-batch latent-denoiser updates.

During generation, DDIM reverse sampling starts from Gaussian latent noise and is conditioned on the target SOC value throughout denoising. The implementation used 50 DDIM reverse steps, equal to the diffusion horizon, with η=0 for deterministic sampling. The resulting synthetic spectral latent vector is decoded together with the SOC label embedding to obtain the generated spectrum. Each generated spectrum is then paired with the same conditioning SOC value to form the labeled pseudo-sample used for downstream regression-model augmentation.

(12) $$x^{syn}=D\left(z_{0}^{syn},\phi \left(y\right)\right)$$

In HsDDIM, “conditional” therefore denotes SOC-guided spectral generation: y influences the denoising trajectory and spectral reconstruction but is not itself a generated variable. The model learns the distribution of spectral latent representations conditional on SOC and reconstructs spectra from sampled latent variables under the specified SOC condition. Consequently, HsDDIM increases spectral diversity for a given SOC domain rather than creating new laboratory reference SOC values.

The principal parameter settings were determined through different mechanisms rather than a single exhaustive grid search. Spectral preprocessing choices and augmentation ratios were evaluated using the comparative experiments reported in the Results section. SVR and XGBoost hyperparameters were selected by five-fold cross-validation within the modeling data. The VAE, GAN, and WGAN-GP baseline settings followed the cited comparative framework [9], whereas the 23-dimensional HsDDIM bottleneck was supported by the preliminary and repeated latent-dimensionality comparison summarized in Supplementary Table S2. The Transformer depth, number of attention heads, model width, diffusion schedule, and DDIM sampling configuration were fixed during model development as stable implementation settings for the small modeling set and were kept unchanged throughout the comparative experiments; they are not claimed to result from exhaustive independent grid searches.

### 2.5. SOC Prediction Models

This study used three models, SVR, XGBoost, and 1D-CNN, to evaluate the effects of data augmentation on SOC inversion performance. SVR is suitable for high-dimensional, small-sample, and nonlinear regression problems and can handle complex spectral responses through kernel mapping [31]. XGBoost models nonlinear relationships by integrating multiple regression trees and has strong robustness [32]. 1D-CNN directly uses one-dimensional spectral sequences as inputs and extracts local response features among adjacent bands using convolutional kernels [33,34].

**Figure 6:**
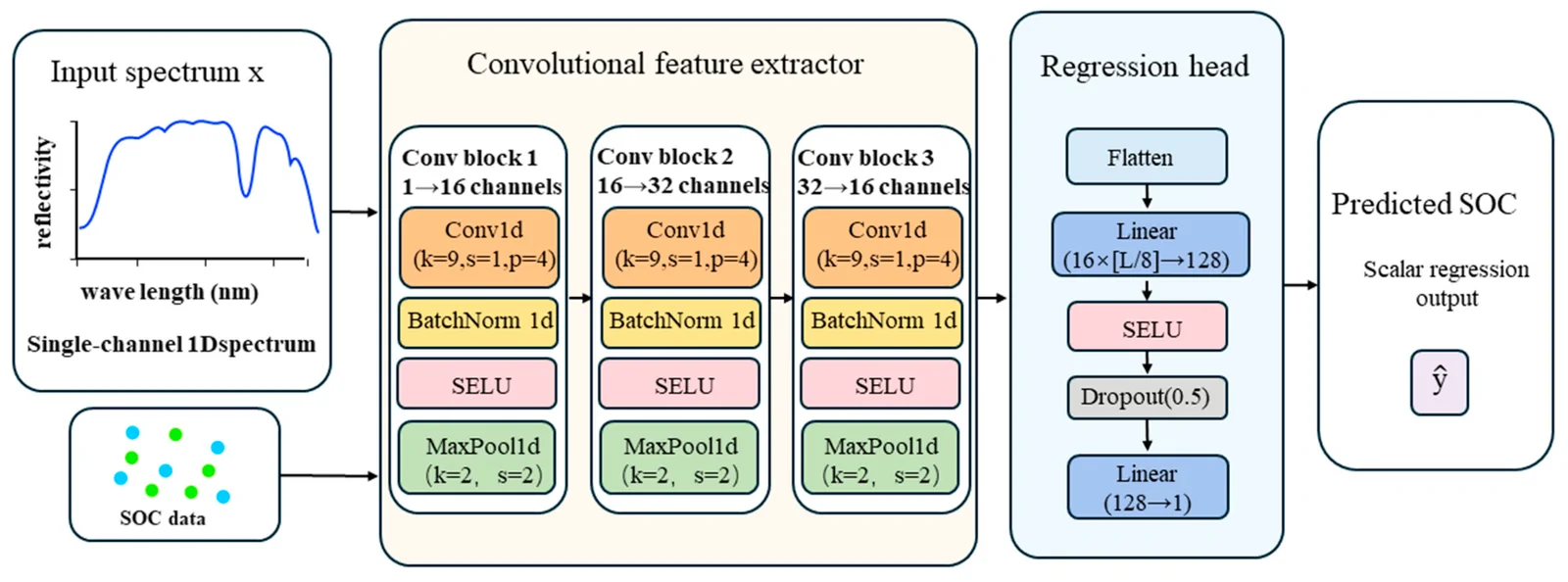
Structure of the 1D-CNN spectral regression model. The convolutional blocks extract local spectral features, and the regression head outputs the predicted SOC value. Arrows indicate the forward information flow through the convolutional feature extractor and regression head, while colors distinguish different layer types and functional components and do not encode quantitative information.

For SVR and XGBoost, model-specific input standardization and hyperparameter optimization were performed within the modeling data, with five-fold cross-validation used for parameter selection. The 1D-CNN comprised three Conv1D-BatchNorm-SELU-MaxPool blocks with 16, 32, and 16 filters. All convolutional layers used a kernel size of 9 and stride of 1, and max pooling used a kernel size and stride of 2. The regression head mapped the flattened feature representation through a fully connected layer, SELU activation, dropout (rate = 0.5), and a final linear layer to the SOC output. The structure of the 1D-CNN spectral regression model is shown in Figure 6. Training was allowed for up to 250 epochs with a batch size of 16. Optimization used Adam with an initial learning rate of 1 × 10^−4^ and SmoothL1Loss (Huber-type loss). For early stopping, 30% of the current training pool was randomly reserved as an internal validation subset using seed 42; the internal validation loss was monitored with a patience of 50 epochs, and the best model state was restored before evaluation on the fixed 74-sample validation set. Thus, the fixed validation set did not control CNN gradient updates or early stopping. To assess run-to-run robustness of the final configuration, the HsDDIM-augmented 1D-CNN at the selected 300% augmentation ratio was evaluated in five additional independent runs using different random seeds, while the data partition, model architecture, and evaluation settings were kept unchanged. The corresponding training and validation metrics were recorded for stability analysis. All computational analyses were implemented in Python 3.10.18 (Python Software Foundation, Beaverton, OR, USA). Deep-learning models were developed using PyTorch 2.2.0 (PyTorch Foundation, Linux Foundation, San Francisco, CA, USA), whereas conventional machine-learning models were implemented using scikit-learn 1.7.1 (The scikit-learn developers) and XGBoost 3.2.0 (XGBoost contributors).

### 2.6. Model Evaluation Metrics

Prediction performance was evaluated using the coefficient of determination (R^2^), ratio of performance to deviation (RPD), and root mean square error (RMSE). RPD was interpreted using commonly applied Vis–NIR soil-spectroscopy criteria [35]: RPD < 1.0 indicates very poor prediction; 1.0 ≤ RPD < 1.4, poor; 1.4 ≤ RPD < 1.8, fair; 1.8 ≤ RPD < 2.0, good; 2.0 ≤ RPD < 2.5, very good; and RPD ≥ 2.5, excellent. RMSE retains the unit of the target variable (g kg^−1^) and depends on the SOC range and variability of the evaluated dataset; therefore, no universal absolute RMSE threshold was imposed. Instead, RMSE was interpreted comparatively within the same fixed validation set, with lower values indicating better prediction relative to the non-augmented baseline and the observed SOC variability. Values of R^2^ closer to 1, higher RPD, and lower RMSE therefore indicate stronger predictive performance. Generated-sample quality was evaluated using generated-spectrum visualization, t-SNE distributions [36], maximum mean discrepancy (MMD) [37], Fréchet Inception Distance (FID) [38], and downstream prediction performance. Lower MMD and FID values indicate closer statistical agreement between generated and real sample distributions, but these metrics do not by themselves establish physical spectral validity. Therefore, generated curves were also interpreted with respect to realistic reflectance ranges, overall spectral morphology, and the major absorption regions observed in the real soil spectra. Downstream prediction performance was used as an additional practical assessment of whether synthetic samples were associated with improved prediction on real samples in the fixed validation set.

For the distributional comparison reported in the Results section, MMD and FID were calculated from the spectral variables rather than the SOC labels, following the same evaluation procedure as the comparative hyperspectral augmentation framework in Xia et al. [9]. For MMD, the squared Gaussian radial-basis-function (RBF) kernel statistic was used. For real spectra drawn from distribution *P* and generated spectra drawn from distribution *Q*, the statistic is defined as follows [37]:

(13) $${MMD}^{2}\left(P,Q\right)=E\left[k\left(x,x^{\prime }\right)\right]+E\left[k\left(g,g^{\prime }\right)\right]-2E\left[k\left(x,g\right)\right]$$

(14) $$k\left(a,b\right)=exp\left(-\gamma {∥a-b∥}^{2}\right),\;\;\gamma =\frac{1}{2\sigma^{2}}$$

where $x,x^{\prime }$ are real spectra, $g,g^{\prime }$ are generated spectra, $k\left(\cdot ,\cdot \right)$ is the Gaussian RBF kernel, and σ is the kernel bandwidth, set to the median distance between the real and generated spectral samples. The squared statistic MMD^2^ is reported under the label “MMD” for consistency with the implemented evaluation pipeline.

FID was computed as a Fréchet distance in a PCA feature space rather than by using an image Inception network. Because direct covariance estimation in the original high-dimensional spectral space can be unstable, the real and generated spectra were first represented in a 20-dimensional PCA space, following the same implementation used in the comparative framework [9]. The FID statistic was then calculated as [38]

(15) $$FID\left(P,Q\right)\;=\;∥\mu_{P}-\mu_{Q}{∥}_{2}^{2}+Tr\left(\Sigma_{P}+\Sigma_{Q}-2{\left(\Sigma_{P}\Sigma_{Q}\right)}^{1/2}\right)$$

where $\mu_{P}$ and $\Sigma_{P}$ are the mean vector and covariance matrix of the real spectra in the 20-dimensional PCA feature space, $\mu_{Q}$ and $\Sigma_{Q}$ are the corresponding quantities for the generated spectra, and Tr denotes the matrix trace. For both MMD and FID, lower values indicate closer statistical agreement between generated and real spectral distributions; neither metric alone establishes physical validity.

### 2.7. Synthetic-Sample Memorization Assessment

Among the evaluated augmentation ratios, the 300% setting yielded the highest observed validation performance for the 1D-CNN and was therefore selected as the final augmentation configuration. To ensure that the memorization assessment corresponded exactly to the synthetic data used in the final predictive experiment, the analysis was conducted on the 522 HsDDIM-generated spectra associated with this selected 300% configuration.

All distance-based analyses were performed in the 201-dimensional preprocessed spectral space; SOC values were not included in the distance calculation. Because the spectral variables were continuous, Euclidean distance was used to quantify the similarity between a synthetic spectrum $x^{syn}$ and a real modeling spectrum $x^{real}$:

(16) $$d\left(x^{syn},x^{real}\right)=\sqrt{\sum_{k=1}^{201}{\left(x_{k}^{syn}-x_{k}^{real}\right)}^{2}}$$

where *k* indexes the 201 preprocessed spectral bands. Exact duplicate detection was performed between the synthetic and real modeling spectra and within the synthetic dataset. For each synthetic spectrum, the nearest-neighbor distance to the 174 modeling spectra was calculated. Two real-sample reference comparisons were also computed: nearest-neighbor distances among the modeling spectra (excluding self-matches) and nearest-neighbor distances from the fixed validation spectra to the modeling spectra. In this memorization diagnostic, the fixed validation samples served only as an empirical real-sample distance reference and were not used for generative-model fitting. Near-identical synthetic spectra were additionally screened using a nearest-neighbor distance threshold of 10^−4^. These two real-sample comparisons were treated as empirical distance scales, rather than as formal upper or lower thresholds that the synthetic samples were required to exceed.

As a complementary relative nearest-neighbor diagnostic, the criterion used by Fang et al. [39] was adopted. For each synthetic spectrum, $d_{1}$ and $d_{2}$ denote the Euclidean distances to its nearest and second-nearest real modeling spectra, respectively. A synthetic spectrum satisfying $\frac{d_{1}}{d_{2}}<\frac{1}{3}$ was flagged as potentially memorized. This relative-distance criterion was used as an additional screening diagnostic and was not treated as evidence of exact duplication by itself.

## 3. Results

### 3.1. SOC Statistical Characteristics and Raw Spectral Response

The SOC content of the 248 study samples ranged from 2.62 to 66.87 g kg^−1^, with a mean of 21.52 g kg^−1^, a standard deviation of 11.93 g kg^−1^, and a coefficient of variation of 55%. The 174-sample modeling set had a mean SOC content of 20.53 g kg^−1^ and a standard deviation of 12.00 g kg^−1^, whereas the fixed 74-sample validation set had a mean of 23.84 g kg^−1^ and a standard deviation of 11.51 g kg^−1^. The comparable standard deviations indicate that both subsets retained broad SOC variability after partitioning. The modeling set covered the full SOC range of the original dataset, while the validation set also retained a broad range from 2.72 to 51.27 g kg^−1^. These statistics indicate substantial variability in measured SOC content and provide a broad response range for model development and validation. The statistical characteristics of SOC content in the whole dataset, modeling set, and fixed validation set are summarized in Table 1. However, chemical variability in SOC does not necessarily imply an equally large degree of spectral heterogeneity; the spectral diversity of the samples must also be considered from the observed reflectance patterns and their inter-sample differences. The SOC distributions of the modeling and validation sets are further compared in Supplementary Figure S1.

**Figure 7:**
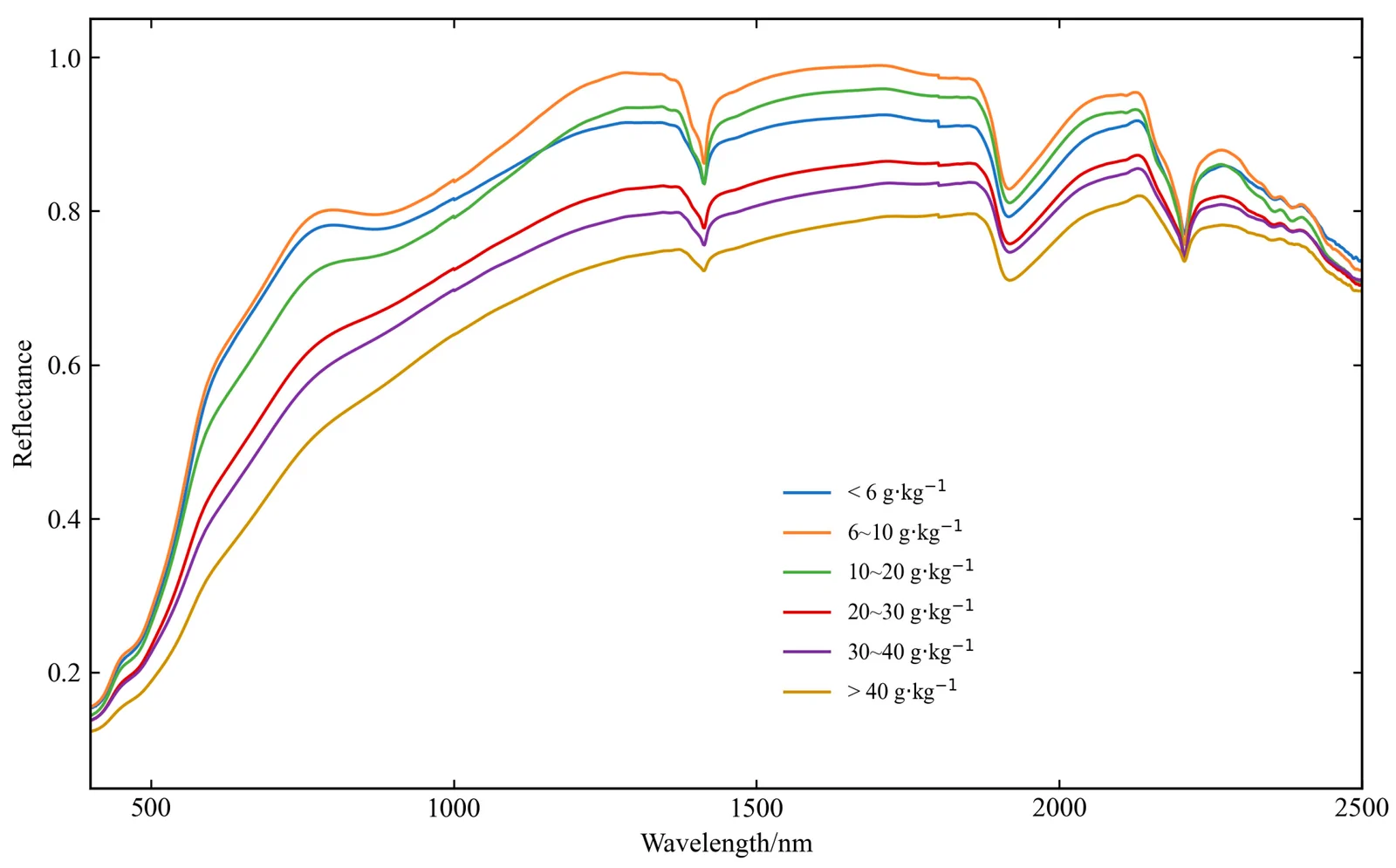
Mean spectral reflectance curves of samples with different SOC levels.

The raw spectra showed typical soil reflectance characteristics: reflectance was low in the visible region, increased rapidly toward the near-infrared region, and exhibited pronounced absorption features near 1400, 1900, and 2200 nm [40,41]. Reflectance varied among samples because the measured signal is jointly affected by SOC, moisture status, iron oxides, clay minerals, particle-size composition, and related soil properties [41]. Overall, the higher-SOC groups tended to exhibit lower reflectance, which is consistent with the darkening and enhanced light absorption associated with organic matter; nevertheless, this tendency should be interpreted as an overall trend rather than a unique SOC-specific spectral response. The mean spectral reflectance curves of samples with different SOC levels are shown in Figure 7.

The mean spectral curves grouped by SOC content further show that samples with different SOC levels shared broadly similar major absorption positions but differed in absorption depth and overall reflectance. All SOC groups exhibited features near 1400, 1900, and 2200 nm. The absorptions around 1400 and 1900 nm are mainly associated with O-H and H_2_O-related vibrations, whereas the feature near 2200 nm may be related to combination vibrations involving Al-OH and Mg-OH groups in clay minerals. These bands are therefore not uniquely attributable to SOC; rather, SOC-related prediction arises from the combined spectral response of organic matter and covarying soil constituents. Although higher-SOC groups generally showed lower overall reflectance, the differences were not strictly monotonic across all wavelength regions, and adjacent SOC groups remained similar in parts of the spectrum. This overlap illustrates why visual interpretation or single-band analysis alone is insufficient and why multivariate machine-learning approaches are useful for extracting distributed spectral information related to SOC.

### 3.2. SOC Prediction Performance Based on Original Samples

Without data augmentation, FD1-SVR performed best on the fixed validation set, with R^2^, RPD, and RMSE values of 0.83, 2.45, and 4.71 g kg^−1^, respectively. The prediction performance of different preprocessing methods and prediction models based on the original samples is summarized in Table 2. According to the RPD categories defined in Section 2.6, the validation RPD of 2.45 corresponds to very good predictive performance. This result suggests that first-order differentiation may have improved SOC-related spectral representation by reducing broad baseline effects and emphasizing local spectral variation. FD2 and FOD preprocessing generally outperformed raw reflectance, but excessively high derivative orders amplified noise and reduced the validation performance of some models.

**Figure 8:**
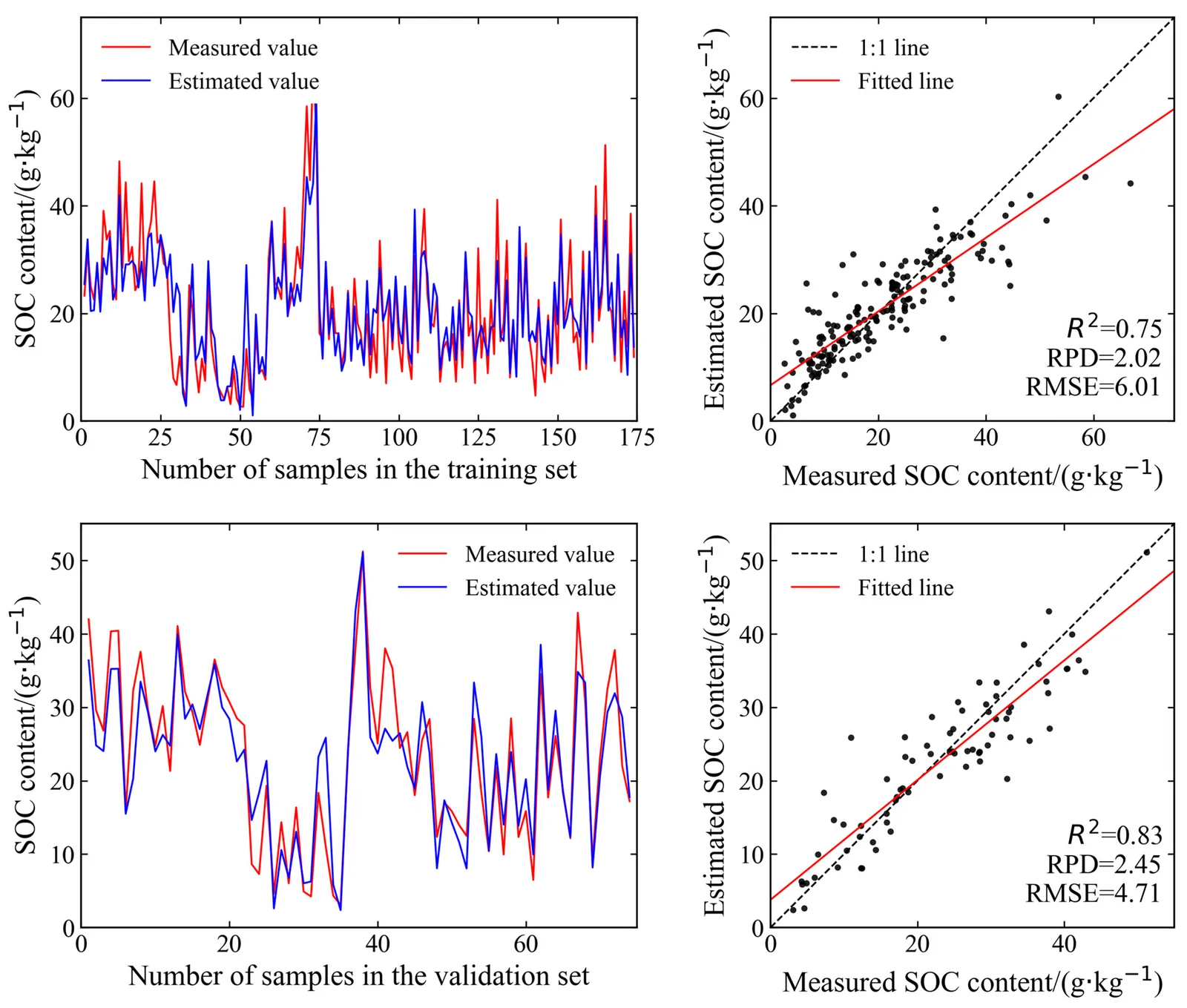
Performance of the best prediction model based on the original samples. Upper row: modeling-set fitting curve (**left**) and measured-versus-predicted scatter plot (**right**); lower row: corresponding fixed-validation-set results.

Under the current sample size and spectral input configuration, XGBoost generally showed lower validation accuracy than SVR across the preprocessing schemes. The 1D-CNN achieved relatively high fitting performance on the FD1 modeling set (R^2^ = 0.84, RPD = 2.50), but its validation performance was lower than that of FD1-SVR (R^2^ = 0.78, RMSE = 5.39 g kg^−1^). The larger training–validation performance gap suggests that 1D-CNN was more susceptible to overfitting under the original small-sample setting. The modeling- and fixed-validation-set performance of the best original-sample model (FD1-SVR) is shown in Figure 8.

### 3.3. Visual and Distributional Consistency Evaluation of Generated Samples

**Figure 9:**
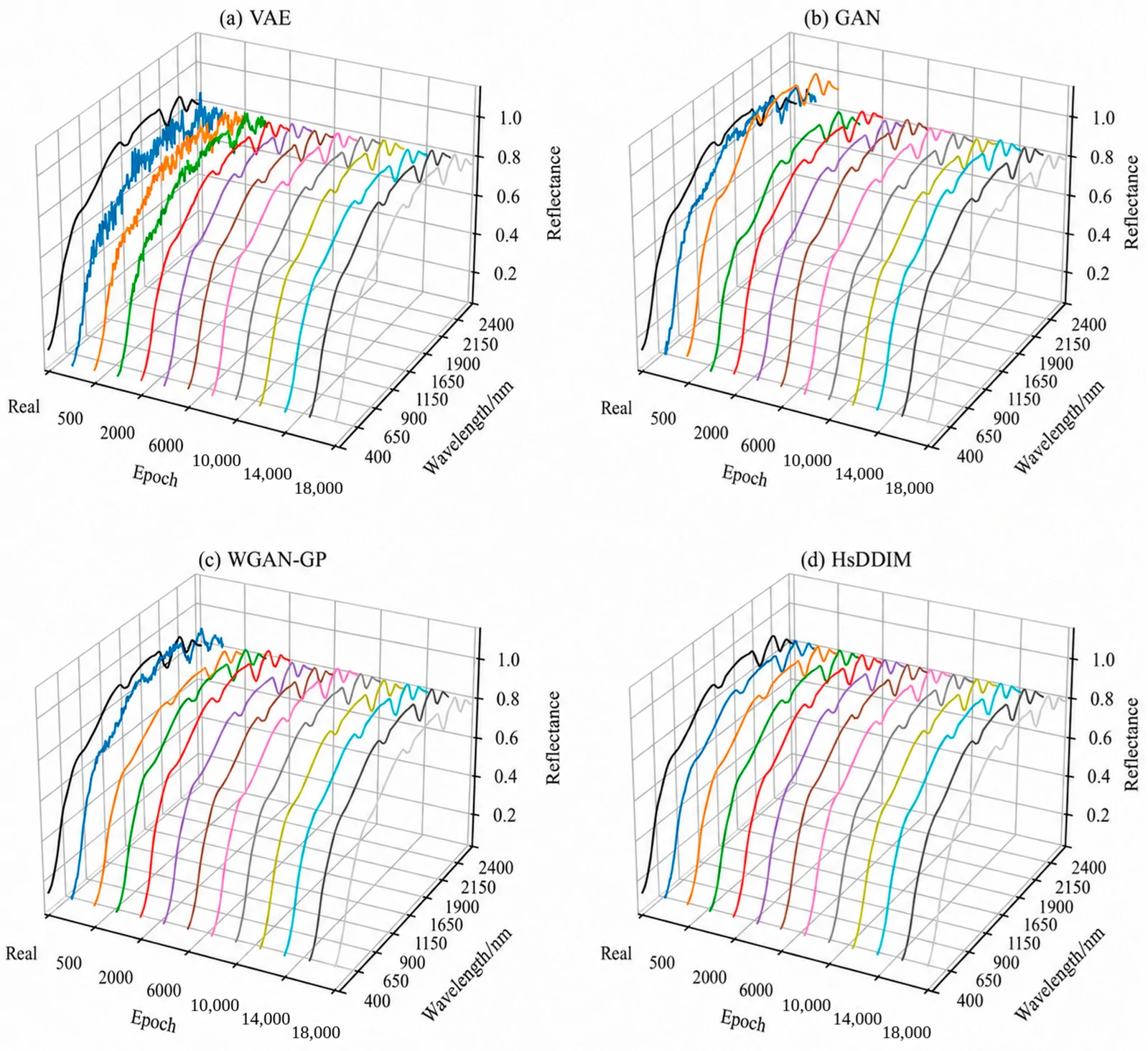
Evolution of generated reflectance curves during training for (**a**) VAE, (**b**) GAN, (**c**) WGAN-GP, and (**d**) HsDDIM. Different colors distinguish spectral curves at different training epochs and do not encode additional quantitative information; the curve labeled “Real” represents the real-sample reference.

As shown in Figure 9, the generated spectral curves progressively became smoother and approached the morphology of the real soil spectra as training proceeded. The principal wavelength-dependent structures and major absorption regions observed in the real spectra were broadly retained. VAE reproduced the overall spectral shape but showed more pronounced fluctuations and weakened local features at some stages, while GAN converged rapidly in the early stage but exhibited reduced diversity and larger distributional deviations later in training. WGAN-GP produced comparatively stable spectral trajectories, whereas HsDDIM showed the closest overall visual agreement with the real samples in spectral morphology, major absorption positions, and overall reflectance range among the models examined. Because visual inspection alone cannot fully establish the validity of generated hyperspectral samples, the spectral-curve comparison was interpreted jointly with t-SNE visualization, SOC-label distribution diagnostics, MMD/FID metrics, and downstream prediction performance.

**Figure 10:**
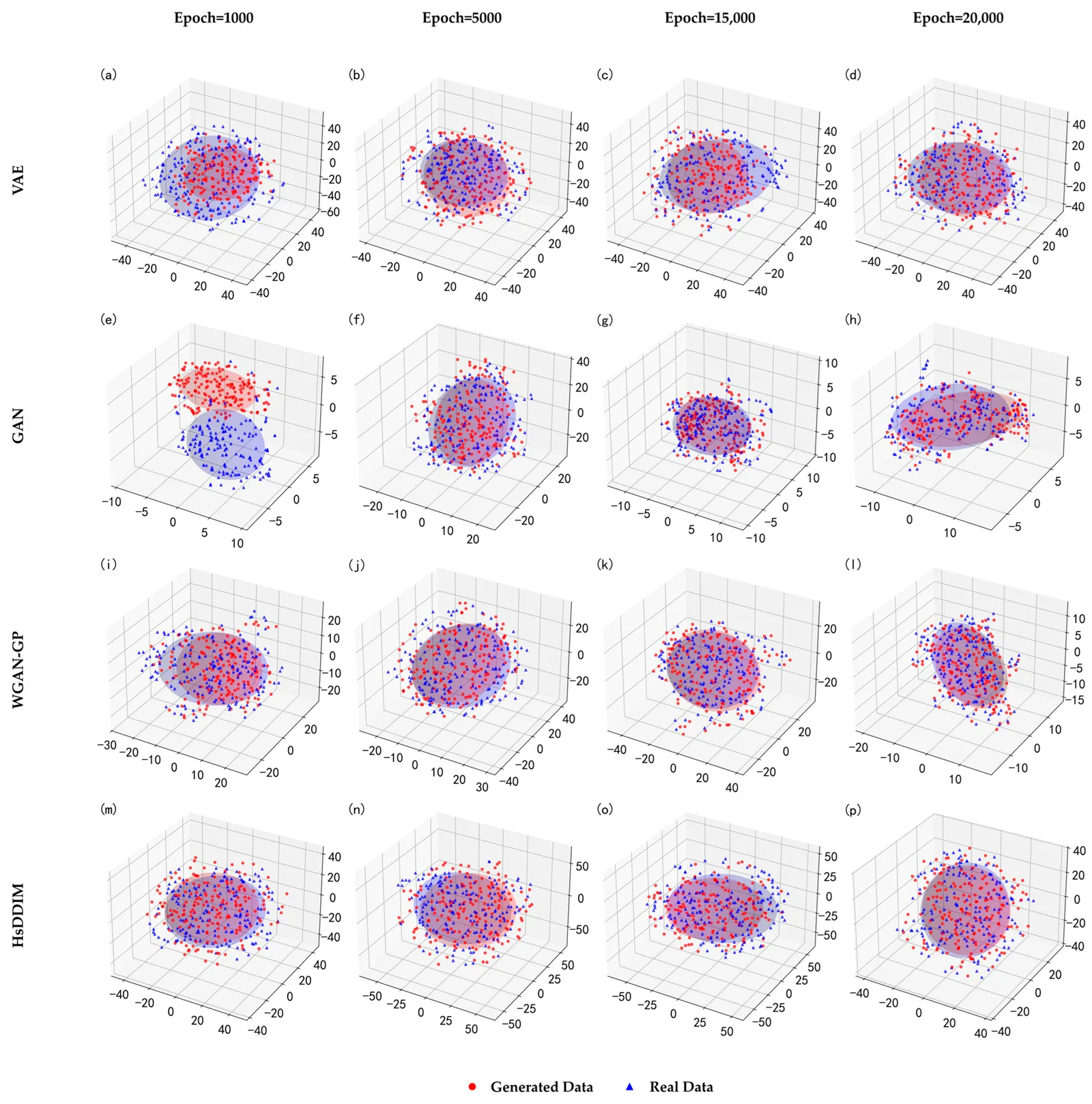
t-SNE distributions of real and generated samples at different training stages. Panels (**a**–**d**), (**e**–**h**), (**i**–**l**), and (**m**–**p**) correspond to VAE, GAN, WGAN-GP, and HsDDIM, respectively; columns show epochs 1000, 5000, 15,000, and 20,000. Red points denote generated samples and blue points denote real samples. Greater overlap indicates closer low-dimensional distributional agreement, but does not by itself establish physical spectral validity.

To further examine spectral distributional agreement between pseudo-samples and real data, t-SNE was used to jointly reduce the dimensionality of real and generated spectral samples (Figure 10). VAE-generated samples showed relatively limited overlap with real samples at several stages, suggesting a degree of distributional shift in latent-space sampling. GAN showed higher overlap at some stages but also visible drift at later stages. WGAN-GP and HsDDIM showed substantial overlap with the real spectral-sample distribution across multiple examined training stages, and HsDDIM exhibited a clustering range, point-cloud density, and center position that were generally closer to those of the real spectral samples. These visual patterns suggest better spectral distributional agreement for HsDDIM, but they are interpreted together with MMD, FID, spectral morphology, and downstream prediction rather than as standalone evidence of generalization.

**Figure 11:**
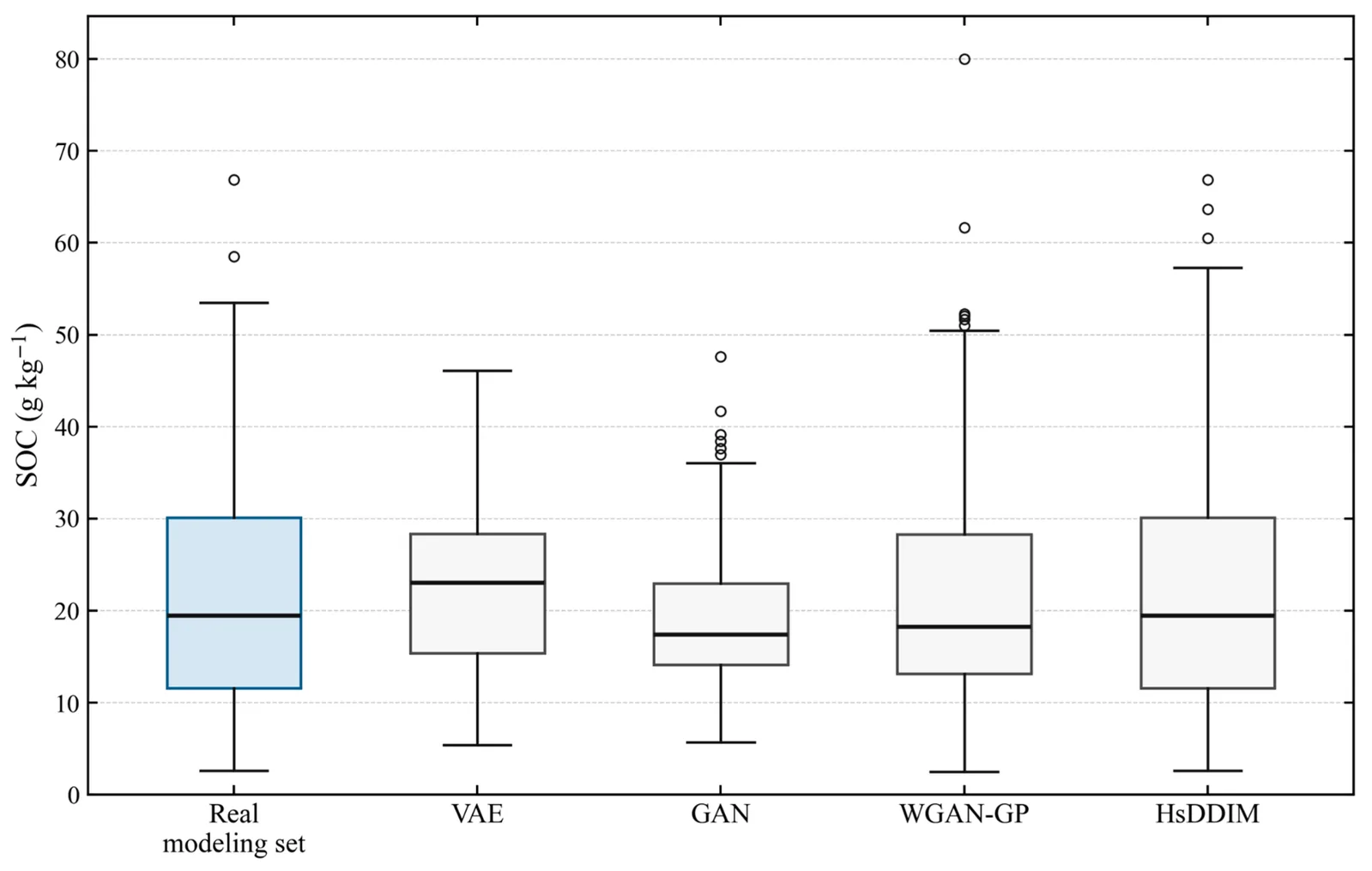
SOC-value distributions associated with the real modeling set and selected stages of VAE (8000 epochs), GAN (14,000), WGAN-GP (16,000), and HsDDIM (19,000). Boxes show Q1–Q3 and the median; whiskers extend to 1.5 × IQR; circles indicate outliers. For HsDDIM, the box summarizes conditioning labels used to guide spectral generation, not independently generated SOC values. The light-blue box denotes the real modeling-set reference, whereas the light-gray boxes denote the SOC-value distributions associated with VAE, GAN, WGAN-GP, and HsDDIM; the colors are used only for visual distinction and do not encode additional quantitative information.

To complement the spectral-space visualization, box plots were used to summarize the SOC values associated with the real modeling set and the selected pseudo-sample sets at the training stages shown in Figure 11. VAE showed an upward shift in the median and a narrower interquartile range (IQR), whereas GAN exhibited the strongest contraction of label variability. WGAN-GP retained a broader SOC-value distribution and showed a median and IQR comparatively close to those of the real modeling set, although several high-SOC outliers remained. HsDDIM must be interpreted differently: its SOC values are conditioning labels supplied to guide spectral generation rather than independently generated outputs. Therefore, the HsDDIM box plot is a diagnostic of the conditioning-label distribution only and should not be interpreted as evidence that HsDDIM generated or reproduced the SOC distribution itself. The corresponding descriptive statistics are provided in Supplementary Table S1 using the same summary indicators as Table 1. Relative to the real modeling-set reference, VAE and GAN showed reduced SOC-value dispersion, whereas WGAN-GP retained a broader range. For HsDDIM, the values in Table S1 describe only the conditioning-label distribution and not independently generated SOC values.

### 3.4. Quantitative Evaluation of Generated Samples

To evaluate the downstream predictive utility of the generated samples, the non-augmented FD1-SVR was used as the baseline for comparison with different augmentation ratios (Table 3). The baseline model achieved R^2^, RPD, and RMSE values of 0.83, 2.45, and 4.71 g kg^−1^ on the fixed validation set, respectively. For VAE and GAN, modeling-set R^2^ increased rapidly as the augmentation ratio increased, but validation improvements were limited. WGAN-GP achieved a low validation RMSE (4.20 g kg^−1^) at a 50% augmentation ratio, but its validation performance fluctuated as the augmentation ratio increased. HsDDIM showed generally improved validation performance with increasing augmentation, although a temporary decline occurred at 250%, and achieved its highest observed validation performance at 300%, with R^2^, RPD, and RMSE values of 0.88, 2.90, and 3.98 g kg^−1^, respectively. Accordingly, the 300% ratio was selected from the tested candidate ratios on the basis of validation performance. It should therefore be interpreted as the best observed validation-selected ratio within the examined range, rather than as a ratio chosen using a separate independent test set. Because the four approaches form labeled pseudo-samples through different generative mechanisms, Table 3 is interpreted as a comparison of augmentation effectiveness under a common downstream evaluation protocol, not as evidence that all four models optimize the same joint spectral-label generation task.

For reporting in Table 4, the listed epochs correspond to representative training stages selected for comparative evaluation by jointly considering the generated-sample diagnostics used in this study, including spectral morphology, t-SNE distribution, MMD/FID behavior, and downstream validation performance. They are therefore referred to as selected training epochs rather than universally optimal checkpoints. The quantitative evaluation results of the four generative models are presented in Table 4. Among the compared models, HsDDIM yielded the lowest MMD (0.0806) and FID (0.5281) at the selected training epoch of 19,000. WGAN-GP ranked second, with an MMD of 0.1058 and an FID of 1.5476 after 16,000 training epochs, whereas GAN obtained values of 0.1481 and 3.6263, respectively. VAE showed the largest MMD and FID values, 0.1855 and 5.2206. Under the present dataset and evaluation settings, this ordering suggests progressively closer statistical agreement with the real hyperspectral sample distribution from VAE and GAN to WGAN-GP and HsDDIM. Because MMD and FID summarize distributional similarity rather than direct physical validity, these results should be interpreted together with the spectral-curve and downstream-prediction analyses.

### 3.5. SOC Prediction Results After HsDDIM Augmentation

HsDDIM was further used to augment the 174 modeling samples at different ratios, and the three prediction models were evaluated on the fixed 74-sample validation set. The prediction performance of SVR, XGBoost, and 1D-CNN under different HsDDIM augmentation ratios is summarized in Table 5. These validation results were used to compare the candidate augmentation ratios and downstream prediction configurations. Overall, data augmentation had a clear positive effect on SVR and 1D-CNN but produced a relatively limited improvement for XGBoost. Under the 300% augmentation ratio, SVR achieved validation R^2^, RPD, and RMSE values of 0.88, 2.90, and 3.98 g kg^−1^, respectively. XGBoost reached its highest observed validation R^2^ (0.86) at the 400% augmentation ratio, whereas 1D-CNN achieved its best validation performance at 300%.

**Figure 12:**
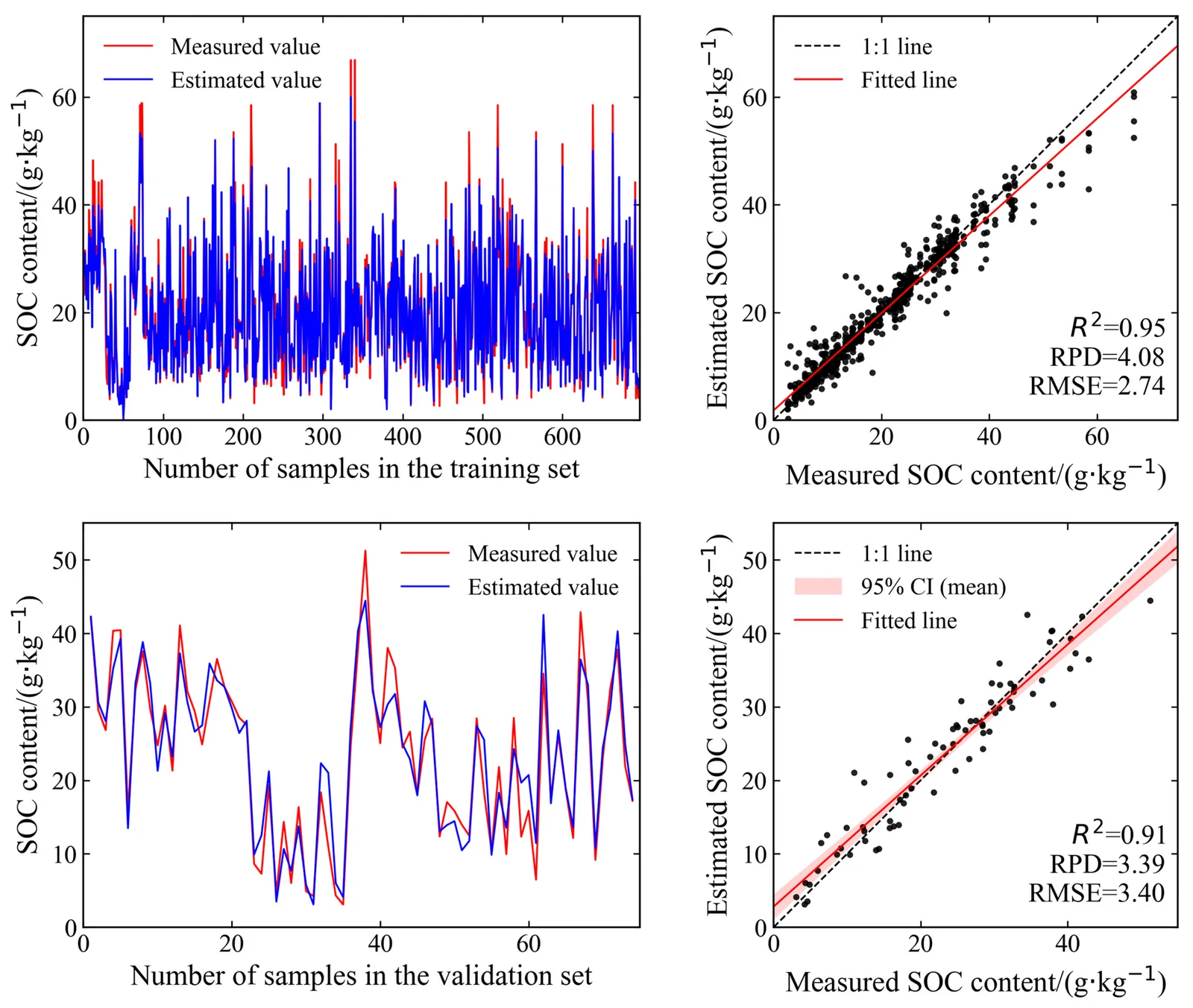
Performance of the final HsDDIM-augmented 1D-CNN. Upper row: augmented modeling-set results; lower row: validation-set results. The shaded band in the lower-right panel denotes the 95% confidence interval of the fitted mean regression line.

The 1D-CNN achieved its highest observed prediction performance at the 300% augmentation ratio, with validation R^2^, RPD, and RMSE values of 0.91, 3.39, and 3.40 g kg^−1^, respectively. Under the adopted RPD criteria, the validation RPD of 3.39 falls in the excellent category. Compared with the non-augmented 1D-CNN (R^2^ = 0.78, RPD = 2.14, RMSE = 5.39 g kg^−1^), R^2^ increased by 0.13, RPD increased by 1.25, and RMSE decreased by 36.9%. Compared with the best model based on the original samples, FD1-SVR (R^2^ = 0.83, RMSE = 4.71 g kg^−1^), R^2^ increased by 0.08 and RMSE decreased by 27.8%. The measured-versus-predicted relationships for this final configuration are shown in Figure 12.

**Figure 13:**
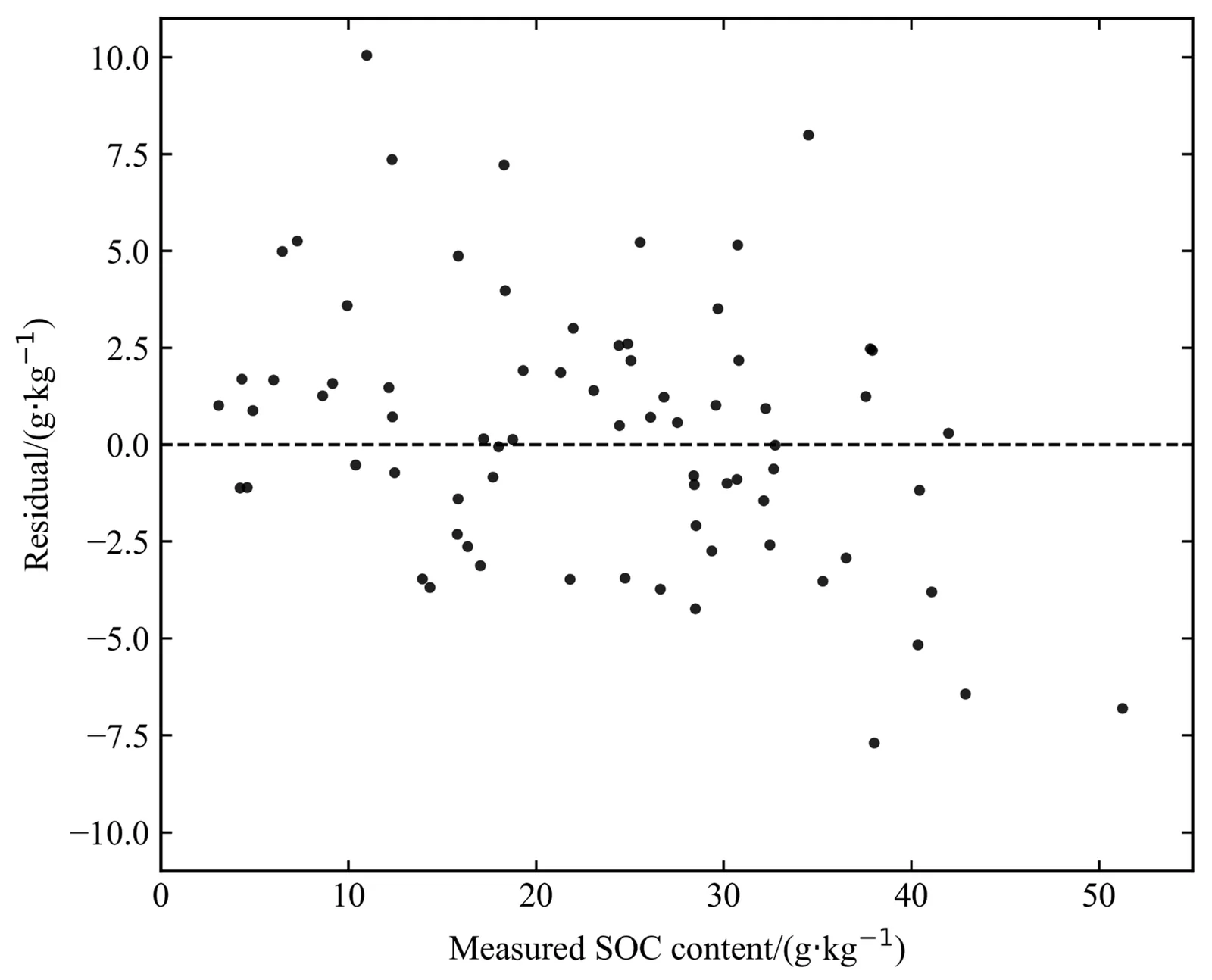
Residual distribution of the final HsDDIM-augmented 1D-CNN on the validation set. Residuals were calculated as predicted SOC minus measured SOC, and the dashed horizontal line denotes zero residual.

Additional diagnostic analysis of the final validation predictions gave a fitted relationship of Predicted = 2.815 + 0.892 × Measured. The fitted slope (0.892) was close to the ideal value of unity. The mean signed prediction bias, calculated as Predicted − Measured, was +0.295 g kg^−1^, and the mean absolute error (MAE) was 2.642 g kg^−1^. As shown in Figure 13, the residuals were generally distributed around zero without a pronounced funnel-shaped pattern, indicating that positive and negative prediction errors were broadly balanced overall. However, negative residuals occurred more frequently toward the higher SOC range, consistent with a slight tendency to underestimate samples with relatively high SOC contents.

### 3.6. Final Model Stability Analysis

To assess the run-to-run stability of the final HsDDIM-augmented 1D-CNN configuration, five additional independent runs were conducted using different random seeds. Table 6 summarizes the corresponding training and validation results.

It can be observed that the validation R^2^, RMSE, and RPD values across the five runs are distributed within relatively narrow ranges, with no evident performance degradation or anomalous runs. The performance gaps between the training and validation sets remain within comparable bounds across runs, and no isolated run shows a marked loss of validation performance. These results indicate that the final HsDDIM-augmented 1D-CNN exhibits good stability and consistency under multiple independent runs, and that its predictive performance is not driven by a single random experiment.

### 3.7. Synthetic-Sample Memorization Analysis

Nearest-neighbor, duplicate-detection, and relative-distance analyses were used to assess whether the selected HsDDIM-generated dataset showed evidence of direct sample replication or excessive proximity to individual modeling spectra. The three nearest-neighbor distance comparisons are summarized in Table 7, while the exact-duplicate, near-identical, and relative nearest-neighbor screening results are reported directly in the accompanying text.

For the selected 300% HsDDIM dataset, the nearest-neighbor distance from the synthetic spectra to the modeling spectra had a mean of 4.998 and a median of 4.969. For reference, the corresponding distances from the fixed validation spectra to the modeling spectra were 3.755 and 3.889, whereas the nearest-neighbor distances among the modeling spectra were 6.193 and 5.890, respectively. Thus, the distance scale for the synthetic spectra was higher than the fixed-validation reference and lower than the within-modeling-set reference; these comparisons were interpreted as empirical real-sample distance scales rather than as a required ordering. No exact duplicates were detected between the 522 synthetic spectra and the 174 real modeling spectra or within the synthetic dataset. The minimum nearest-neighbor distance from the synthetic spectra to the modeling spectra was 1.632, and no synthetic spectrum had a distance below 10^−4^. Under the complementary relative nearest-neighbor screening criterion $\frac{d_{1}}{d_{2}}<\frac{1}{3}$, only 1 of 522 synthetic spectra (0.192%) was flagged as potentially memorized. Taken together, these results provide no evidence of direct sample replication and indicate a very low incidence of relative nearest-neighbor memorization in the selected synthetic dataset.

## 4. Discussion

### 4.1. Effects of Spectral Preprocessing on SOC Inversion

The spectral response of forest red soils reflects the combined influence of SOC, iron oxides, moisture-related O-H absorption, clay minerals, particle size, and scattering effects [41]. In the present data, higher-SOC groups generally exhibited lower overall reflectance, while the major features near 1400, 1900, and 2200 nm remained broadly shared across SOC groups. This pattern is consistent with SOC-related darkening superimposed on absorption features that are also controlled by water- and clay-related constituents. FD1 preprocessing improved predictive performance for several models by emphasizing local slope and shape variation while reducing broad baseline effects. In contrast, FD2 and higher-order FOD transformations can amplify high-frequency noise together with useful local information, which provides a plausible explanation for why increasingly high derivative orders did not produce monotonic improvements across all models.

### 4.2. Interpretation of HsDDIM Augmentation Performance

Across the evaluated generative models, HsDDIM showed the closest overall agreement with the real data according to the combined evidence from spectral morphology, t-SNE patterns, MMD/FID, and downstream prediction results. These complementary indicators are important because no single metric fully characterizes the quality of synthetic hyperspectral samples. The visual comparison in Figure 9 showed that the generated spectra broadly retained the wavelength-dependent structure and major absorption regions of the real soil spectra, while the lower MMD and FID values indicated closer statistical distributional agreement. Together with the improved downstream prediction performance, these observations support latent diffusion as a promising augmentation strategy for the present small-sample SOC dataset. However, its effectiveness beyond the current soil type, sensor configuration, and experimental protocol remains to be evaluated.

A key feature of HsDDIM is that the diffusion process operates in the spectral latent space while SOC is supplied as an explicit conditioning signal to both denoising and decoding. In probabilistic terms, the intended task is conditional spectral generation given SOC rather than joint stochastic generation of spectra and SOC. The practical role of HsDDIM is therefore to increase spectral diversity under specified SOC conditions and to pair each generated spectrum with the SOC value that guided its generation. This design is intended to reduce inconsistency between generated spectra and their associated SOC labels without treating SOC as a stochastic diffusion dimension. The improved downstream prediction observed with HsDDIM is consistent with, but does not by itself prove, better preservation of the spectral-label relationship. Additional checks of physical absorption characteristics, cross-region datasets, and other sensors will be important for determining how well this relationship transfers beyond the current laboratory setting.

Compared with adversarial training, the noise-prediction objective used by diffusion models avoids direct generator–discriminator competition and may reduce sensitivity to mode-collapse-type behavior. In the present experiments, GAN and WGAN-GP showed more visible variation across training stages, whereas HsDDIM produced comparatively stable spectral morphology and distributional metrics at the selected stages. These findings are specific to the current dataset, hyperparameter settings, and evaluation protocol and should not be interpreted as evidence that diffusion-based augmentation will always outperform adversarial alternatives. In addition, the five-run stability analysis of the final HsDDIM-augmented 1D-CNN showed limited variation in validation performance, providing further evidence that the observed predictive accuracy was not attributable to a single random run.

### 4.3. Effects of Augmentation Ratio on Prediction Performance

The results indicate that increasing the amount of synthetic data does not necessarily produce monotonic gains [9]. In this study, 1D-CNN reached its highest observed validation performance at the 300% augmentation ratio, followed by lower performance at 400–800%. A similar increase-then-decrease pattern with a 300% peak has been reported in hyperspectral soil data augmentation [9]. One possible explanation is that moderate augmentation supplements under-represented regions of the modeling distribution, whereas excessive pseudo-sample proportions may progressively amplify small distributional mismatches between generated and real spectra. Importantly, the 300% ratio was selected based on performance on the fixed validation set and should therefore be regarded as the best-performing ratio within the tested range rather than as a universally optimal augmentation setting.

### 4.4. Limitations and Future Research

This study still has several limitations. First, all samples were collected from a single forest red-soil region in Guangxi, and HsDDIM has not yet been evaluated on established public soil-spectral benchmark datasets. Public spectral libraries may differ from the present dataset in soil type, geographic origin, spectral acquisition system, wavelength configuration, sample preparation, preprocessing procedure, and SOC reference analytical protocol, introducing substantial domain shifts. The present findings should therefore be interpreted within the current geographic, pedological, sensor, and laboratory setting rather than as evidence of universal cross-domain performance. Future work should evaluate HsDDIM on established public soil spectral libraries and multi-regional datasets, including cross-domain and cross-instrument settings [42,43]. Second, the spectral data were laboratory measurements of prepared soil samples. Transfer to field-measured spectra, different sensor platforms or spectral resolutions, and unmanned aerial vehicle or satellite observations may introduce additional effects from moisture, illumination, atmosphere, vegetation cover, and sensor response functions [40,41,44]. Third, although the additional duplicate-detection and nearest-neighbor analyses found no direct sample replication and the complementary relative nearest-neighbor screening identified only a very low proportion of potentially memorized samples, these checks cannot exclude all possible forms of generative-model memorization. Future work should therefore evaluate sample novelty using larger external datasets and complementary feature-space or density-based measures, together with more explicit physical-spectral validation of SOC-related absorption behavior. Fourth, the present comparison focused on learned generative models for spectral data augmentation and did not include simple perturbation-based methods, such as noise addition, shifting, or interpolation, as formal baselines; evaluating these methods under the same data-splitting protocol would provide a useful additional reference. Finally, because HsDDIM conditions spectral generation on SOC rather than generating SOC itself, the present framework primarily expands spectral variability within the modeled SOC domain; extrapolation to previously unseen SOC ranges was not evaluated. The current HsDDIM formulation can be extended to incorporate soil type, topographic factors, stand structure, or sampling depth as additional information. Future studies should evaluate such extensions using independent external datasets to determine their cross-regional transferability.

## 5. Conclusions

This study proposed and evaluated an HsDDIM-based latent conditional diffusion data augmentation strategy for small-sample hyperspectral prediction of SOC in forest red soils. The main conclusions are as follows:

(1) The SOC content of the 248 study samples ranged from 2.62 to 66.87 g kg^−1^, with a coefficient of variation of 55%, indicating substantial variability in measured SOC content. The spectral curves showed common absorption features near 1400, 1900, and 2200 nm, while higher-SOC groups generally exhibited lower overall reflectance.
(2) Without data augmentation, FD1-SVR achieved the best validation performance (R^2^ = 0.83, RPD = 2.45, RMSE = 4.71 g kg^−1^), indicating that first-order differentiation combined with kernel regression showed strong predictive performance under the current small-sample condition.
(3) Among the four learned augmentation models, HsDDIM yielded the lowest MMD and FID values, 0.0806 and 0.5281, respectively. In the FD1-SVR augmentation comparison, the 300% HsDDIM ratio achieved a validation RMSE of 3.98 g kg^−1^. Under the present dataset and evaluation settings, these results indicate closer statistical agreement between HsDDIM-generated and real spectra than for the other compared augmentation strategies. HsDDIM generates spectra under specified SOC conditions rather than generating SOC values themselves. Memorization analysis of the final 300% synthetic dataset detected no exact synthetic–modeling duplicates, and only 1 of 522 synthetic spectra (0.192%) was flagged by the complementary relative nearest-neighbor screening criterion, indicating a very low observed incidence of potential relative memorization in the selected generated dataset.
(4) HsDDIM augmentation improved the observed SOC prediction performance of 1D-CNN in the present experiments. At the 300% augmentation ratio, 1D-CNN achieved R^2^ = 0.91, RPD = 3.39, and RMSE = 3.40 g kg^−1^ on the fixed validation set, representing the best observed result under the present protocol. Performance decreased at higher augmentation ratios, indicating that generation quality and pseudo-sample proportion should be considered jointly rather than assuming that more synthetic data will always improve prediction. Across five independent runs, the validation metrics remained within relatively narrow ranges, indicating that the final model performance was not driven by an isolated random run.

Further validation on established public soil-spectral benchmark datasets and multi-regional samples is required to determine the broader transferability of HsDDIM across different soil types, regions, and sensing systems.

## Figures and Tables

**Table 1:** Statistical characteristics of SOC content in the whole dataset, modeling set, and fixed validation set.

Dataset	No. of Samples	Minimum (g kg^−1^)	Maximum (g kg^−1^)	Mean (g kg^−1^)	SD	CV (%)
Whole set	248	2.62	66.87	21.52	11.93	55
Modeling set	174	2.62	66.87	20.53	12.00	58
Fixed validation set	74	2.72	51.27	23.84	11.51	48

**Table 2:** SOC prediction performance of different preprocessing methods and prediction models based on original samples. RPD and RMSE interpretation follows the criteria defined in Section 2.6. RMSE is expressed in g kg^−1^.

Model	Spectral Processing	Training R^2^	Training RPD	Training RMSE	Validation R^2^	Validation RPD	Validation RMSE
SVR	Raw	0.51	1.44	8.44	0.60	1.59	7.10
XGBoost		0.46	1.36	8.82	0.45	1.36	8.49
1D-CNN		0.59	1.57	7.63	0.40	1.30	8.84
SVR	FD1	0.75	2.02	6.01	0.83	2.45	4.71
XGBoost		0.72	1.89	6.34	0.75	2.02	5.70
1D-CNN		0.84	2.50	4.81	0.78	2.14	5.39
SVR	FD2	0.77	2.11	5.77	0.81	2.30	5.02
XGBoost		0.74	1.95	6.15	0.70	1.84	6.26
1D-CNN		0.75	2.01	5.96	0.64	1.69	6.81
SVR	1.2-FOD	0.77	2.09	5.82	0.81	2.31	4.98
XGBoost		0.70	1.83	6.57	0.72	1.91	6.03
1D-CNN		0.77	2.07	5.80	0.67	1.76	6.56
SVR	1.4-FOD	0.75	2.02	6.02	0.79	2.18	5.29
XGBoost		0.70	1.82	6.59	0.75	2.00	5.77
1D-CNN		0.77	2.11	5.70	0.67	1.74	6.61
SVR	1.6-FOD	0.75	2.00	6.09	0.78	2.15	5.34
XGBoost		0.74	1.96	6.13	0.70	1.84	6.27
1D-CNN		0.79	2.21	5.44	0.70	1.84	6.25
SVR	1.8-FOD	0.76	2.04	5.95	0.80	2.24	5.15
XGBoost		0.73	1.93	6.22	0.75	2.01	5.74
1D-CNN		0.80	2.22	5.40	0.69	1.81	6.36

**Table 3:** Downstream FD1-SVR performance under different generative models and augmentation ratios. RPD and RMSE interpretation follows the criteria defined in Section 2.6. RMSE is expressed in g kg^−1^.

Generation Model	Ratio	Training R^2^	Training RPD	Training RMSE	Validation R^2^	Validation RPD	Validation RMSE
No augmentation	0%	0.75	2.02	6.01	0.83	2.45	4.71
VAE	50%	0.89	3.07	3.24	0.82	2.41	4.78
	100%	0.92	3.51	2.45	0.83	2.50	4.61
	150%	0.93	3.84	2.00	0.84	2.54	4.52
	200%	0.94	4.24	1.66	0.84	2.55	4.51
	250%	0.96	4.73	1.38	0.84	2.58	4.47
	300%	0.96	5.19	1.17	0.84	2.58	4.46
GAN	50%	0.89	3.01	4.29	0.84	2.56	4.50
	100%	0.91	3.33	3.90	0.84	2.55	4.51
	150%	0.93	3.72	3.45	0.85	2.59	4.44
	200%	0.94	4.05	3.27	0.85	2.60	4.43
	250%	0.95	4.50	3.03	0.85	2.61	4.41
	300%	0.96	5.21	2.66	0.85	2.61	4.42
WGAN-GP	50%	0.86	2.68	4.29	0.84	2.74	4.20
	100%	0.88	2.89	3.81	0.85	2.51	4.58
	150%	0.89	3.04	3.55	0.86	2.62	4.40
	200%	0.89	3.02	3.61	0.85	2.68	4.30
	250%	0.90	3.17	3.41	0.85	2.64	4.35
	300%	0.91	3.27	3.30	0.86	2.64	4.36
HsDDIM	50%	0.82	2.38	4.77	0.85	2.58	4.45
	100%	0.84	2.47	4.67	0.86	2.71	4.25
	150%	0.88	2.87	4.07	0.87	2.81	4.10
	200%	0.87	2.75	4.18	0.87	2.80	4.11
	250%	0.90	3.10	3.90	0.84	2.56	4.50
	300%	0.91	3.31	3.42	0.88	2.90	3.98

**Table 4:** MMD and FID evaluation results of the four generative models at the selected training stage. Lower MMD and FID values indicate closer statistical agreement with the real-sample distribution.

Model	Selected Training Epoch	MMD	FID
VAE	8000	0.1855	5.2206
GAN	14,000	0.1481	3.6263
WGAN-GP	16,000	0.1058	1.5476
HsDDIM	19,000	0.0806	0.5281

**Table 5:** SOC prediction performance of SVR, XGBoost, and 1D-CNN after HsDDIM data augmentation at different ratios. RPD and RMSE interpretation follows the criteria defined in Section 2.6. RMSE is expressed in g kg^−1^.

Augmentation Set	Model	Training R^2^	Training RPD	Training RMSE	Validation R^2^	Validation RPD	Validation RMSE
174 + 0% (0)	SVR	0.75	2.02	6.01	0.83	2.45	4.71
	XGBoost	0.73	1.93	6.22	0.75	2.01	5.74
	1D-CNN	0.84	2.50	4.81	0.78	2.14	5.39
174 + 50% (87)	SVR	0.82	2.38	4.77	0.85	2.58	4.45
	XGBoost	0.73	1.94	6.40	0.80	2.27	5.08
	1D-CNN	0.88	2.95	4.29	0.84	2.55	4.51
174 + 100% (174)	SVR	0.84	2.47	4.67	0.86	2.71	4.25
	XGBoost	0.75	2.02	6.06	0.82	2.43	4.73
	1D-CNN	0.87	2.78	4.10	0.85	2.67	4.31
174 + 150% (261)	SVR	0.88	2.87	4.07	0.87	2.81	4.10
	XGBoost	0.77	2.07	5.89	0.83	2.48	4.63
	1D-CNN	0.88	2.95	3.86	0.87	2.87	4.01
174 + 200% (348)	SVR	0.87	2.75	4.18	0.87	2.80	4.11
	XGBoost	0.73	1.94	6.06	0.83	2.47	4.65
	1D-CNN	0.87	2.83	3.90	0.89	3.04	3.78
174 + 250% (435)	SVR	0.90	3.10	3.90	0.84	2.56	4.50
	XGBoost	0.79	2.18	5.60	0.85	2.63	4.37
	1D-CNN	0.95	4.22	2.71	0.90	3.30	3.49
174 + 300% (522)	SVR	0.91	3.31	3.42	0.88	2.90	3.98
	XGBoost	0.76	2.06	6.06	0.84	2.58	4.46
	1D-CNN	0.95	4.08	2.74	0.91	3.39	3.40
174 + 400% (696)	SVR	0.93	3.67	3.37	0.86	2.71	4.25
	XGBoost	0.86	2.66	4.66	0.86	2.74	4.20
	1D-CNN	0.92	3.60	3.31	0.89	3.05	3.78
174 + 600% (1044)	SVR	0.93	3.83	3.05	0.87	2.83	4.06
	XGBoost	0.82	2.36	5.14	0.85	2.69	4.29
	1D-CNN	0.94	4.04	3.00	0.87	2.88	4.00
174 + 800% (1392)	SVR	0.96	4.79	2.59	0.86	2.79	4.13
	XGBoost	0.88	2.94	4.22	0.85	2.69	4.27
	1D-CNN	0.92	3.64	3.29	0.85	2.66	4.33

**Table 6:** Training and validation results of the HsDDIM-augmented 1D-CNN across five additional independent runs. RPD and RMSE interpretation follows the criteria defined in Section 2.6. RMSE is expressed in g kg^−1^.

Run	Training			Validation		
	R^2^	RPD	RMSE	R^2^	RPD	RMSE
1	0.96	5.30	2.28	0.91	3.35	3.43
2	0.96	4.91	2.45	0.91	3.47	3.32
3	0.95	4.63	2.69	0.90	3.29	3.50
4	0.96	5.23	2.37	0.92	3.55	3.25
5	0.97	5.62	2.22	0.90	3.23	3.56

**Table 7:** Nearest-neighbor distance statistics for the selected HsDDIM dataset at the 300% augmentation ratio (522 synthetic spectra), calculated in the 201-dimensional preprocessed spectral space.

Evaluated Spectra	Reference Spectra	Mean	Median	Minimum
Modeling spectra	Modeling spectra *	6.193	5.890	4.684
Fixed validation spectra	Modeling spectra	3.755	3.889	2.135
Synthetic spectra	Modeling spectra	4.998	4.969	1.632

Note: * Self-matches were excluded when calculating nearest-neighbor distances among the modeling spectra.

## Data Availability

The data used in this study are not publicly available as they are internal datasets of the research group. The data may be made available from the corresponding author upon reasonable request.

## Supplementary Materials

The following supporting information can be downloaded at https://www.mdpi.com/article/10.3390/s26175657/s1, Table S1. Statistical characteristics of SOC values associated with the real modeling set and selected pseudo-sample sets used for the distributional comparison in Figure 11; Table S2. Sensitivity of CVAE spectral reconstruction to latent dimensionality and corresponding PCA cumulative explained variance; Figure S1. Box plots of SOC distributions for the modeling set and validation set.
