# Supplementary material for: Latent Conditional Diffusion-Based Data Augmentation for Small-Sample Hyperspectral Prediction of Forest Soil Organic Carbon
Source: Sensors (Basel). 2026 Sep 5;26(17):5657. doi: 10.3390/s26175657 (PMC13568218; doi:10.3390/s26175657)
Supplement: Supplementary file 1 [file sensors-26-05657-s001.zip › sensors-4444710-supplementary.pdf]

Supplementary Materials

Latent Conditional Diffusion-Based Data Augmentation for Small-Sample Hyperspectral Prediction of Forest Soil

Organic Carbon Jian Tang, Weilin Li, Yuanyuan Shi, Yun Deng, and Junyu Zhao

Table S1. Statistical characteristics of SOC values associated with the real modeling set and selected pseudo-sample sets used for the distributional comparison in Figure 11.

Data source	N	Minimum ($g\ kg^{-1}$)	Maximum ($g\ kg^{-1}$)	Mean ($g\ kg^{-1}$)	SD ($g\ kg^{-1}$)	CV (%)
Real modeling set	174	2.62	66.87	20.53	12.00	58.00
VAE	174	5.39	46.12	22.61	8.26	36.52
GAN	174	5.69	47.59	18.97	7.27	38.33
WGAN-GP	174	2.53	80.02	21.15	12.08	57.13
HsDDIM conditioning labels	174	2.62	66.87	21.78	13.05	59.93

Note: Statistics for the real modeling set are reproduced from Table 1 of the main manuscript and are included as the reference row. Statistics for VAE, GAN, WGAN-GP, and HsDDIM correspond to the selected 174-sample sets used for the distributional comparison in Figure 11. For HsDDIM, SOC values are conditioning labels supplied to guide spectral generation rather than independently generated outputs. The 174-sample selected-stage statistics in this table should not be confused with the downstream augmentation ratios in Table 5 (e.g., 522 synthetic spectra at 300% augmentation).

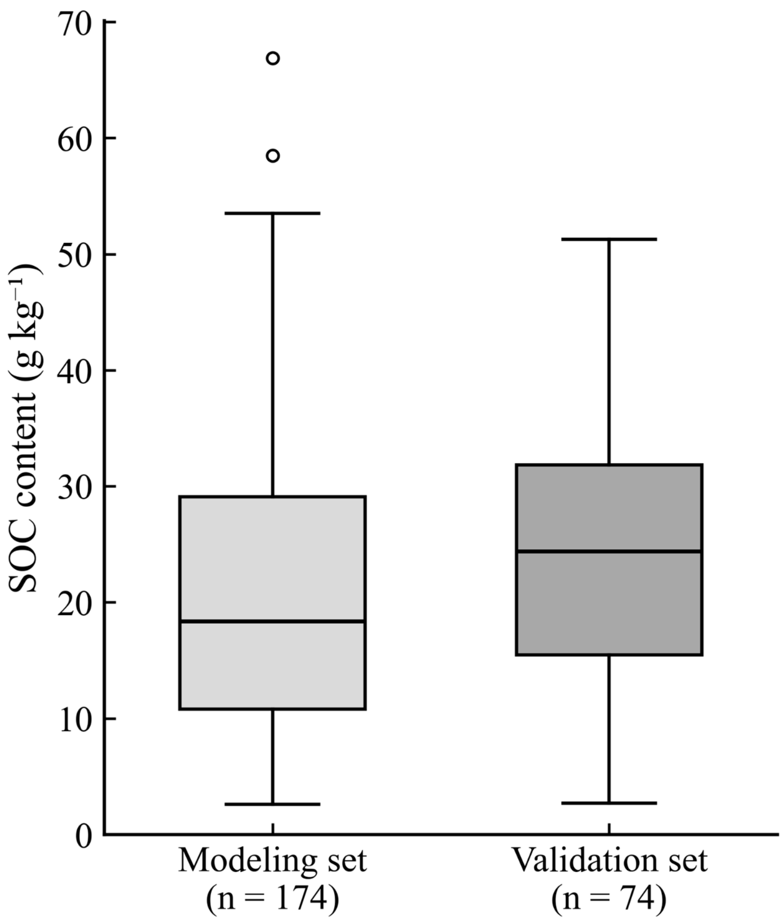

Figure S1. Box plots of SOC distributions for the modeling set (n = 174) and fixed validation set (n = 74). Boxes represent the interquartile range, center lines indicate the medians, whiskers extend to 1.5 × IQR, and circles denote outliers.

Table S2. Sensitivity of CVAE spectral reconstruction to latent dimensionality and corresponding PCA cumulative explained variance.

Latent dimension	Train MSE	Train RMSE	Validation MSE	Validation RMSE	PCA cumulative variance (%)
8	0.000486	0.02205	0.019865	0.14094	95.6238
16	0.000439	0.02095	0.013760	0.11730	99.6224
**23**	0.000726	0.02694	**0.009645**	**0.09821**	99.9235
32	0.000522	0.02286	0.010134	0.10067	99.9879

Note: Reconstruction errors were calculated in the standardized 201-dimensional SG first-derivative spectral space using deterministic posterior-mean reconstruction ($z = \mu$). Latent dimensions of 8, 16, 23, and 32 were evaluated under the same preprocessing and CVAE training configuration; only the spectral latent dimensionality was varied. The lowest validation reconstruction errors are shown in bold.

PCA diagnostic: PCA was fitted only to the 174 modeling samples using the same standardized 201-dimensional SG first-derivative spectra. The Kaiser criterion (eigenvalue > 1) retained 11 components (98.43% cumulative variance). The 95%, 99%, 99.5%, and 99.9% cumulative-variance thresholds required 8, 13, 16, and 22 components, respectively.

Interpretation: The 23-dimensional bottleneck retained 99.9235% of the linear spectral variance and yielded the lowest fixed-validation reconstruction RMSE (0.09821). Increasing the latent dimension to 32 did not improve reconstruction performance (validation RMSE = 0.10067).
